# Author Correction: Human cerebrospinal fluid net flow enhanced by respiration during the awake state

**DOI:** 10.1038/s41467-026-69399-9

**Published:** 2026-02-05

**Authors:** Seokbeen Lim, Petrice M. Cogswell, David N. Jacobson, Mahathi Kandimalla, Yurim Choi, Marin Nycklemoe, Daniel C. Kim, Pragalv Karki, Phillip J. Rossman, Zona McKenzie, John G. Park, Sangwon Lee, Sandeep Ganji, Walter K. Kremers, Ian T. Mark, Daehun Kang, Myung-Ho In, Jeffrey Gunter, Jonathan Graff-Radford, Eduardo E. Benarroch, John Huston, Clifford R. Jack, Ronald C. Petersen, Maria I. Lapid, Val J. Lowe, Daehyun Jung, Paul H. Min

**Affiliations:** 1 Department of Radiology, Mayo Clinic, Rochester, MN USA; 2 Department of Pulmonary and Critical Care Medicine, Mayo Clinic, Rochester, MN USA; 3 Seokmun Domun, Suwon, Republic of Korea; 4 Philips Healthcare, MR R&D, Rochester, MN USA; 5 Quantitative Health Sciences, Mayo Clinic, Rochester, MN USA; 6 Department of Neurology, Mayo Clinic, Rochester, MN USA; 7 Department of Psychiatry and Psychology, Mayo Clinic, Rochester, MN USA; 8 Department of Physiology and Biomedical Engineering, Mayo Clinic, Rochester, MN USA

**Keywords:** Neurophysiology, Respiration, Magnetic resonance imaging

Correction to: *Nature Communications* 10.1038/s41467-025-66548-4, published online 13 December 2025

In the version of this article initially published, there were several typographical errors, inconsistencies in statistics, and errors in reporting. The errors were minor and do not affect the results, interpretation or conclusion of the study. For transparent comparison, a list of the changes is available as Supplementary information accompanying this amendment. The errors have been corrected in the HTML and PDF versions of the article.

## Supplementary information

List of edits to original article

