# Supplementary material for: Human cerebrospinal fluid net flow enhanced by respiration during the awake state
Source: Nat Commun. 2025 Dec 13;16:11499. doi: 10.1038/s41467-025-66548-4 (PMC12749403; doi:10.1038/s41467-025-66548-4)
Supplement: Supplementary file 1 — Supplementary Information [file 41467_2025_66548_MOESM1_ESM.pdf]

Table S1. Demographics

Demographic	T (n=20)	NT (n=25)	Difference (p-value*)
Age			p=0.128
N (Nmiss)	20 (0)	25 (0)	
Mean (SD)	58 (17.3)	49 (20.2)	
Min - Max	29 - 81	21 - 82	
Sex			p=0.764
Female	8 (40%)	12 (48%)	
Male	12 (60%)	13 (52%)	
Systolic Blood Pressure			p=0.457
Mean (SD)	122.35 (9.30)	119.48 (14.91)	
Min - Max	104 - 143	97 - 159	
Diastolic Blood Pressure			p=0.140
Mean (SD)	74.15 (7.07)	78.36 (10.81)	
Min - Max	58 - 90	58 - 108	
Heart Rate, Regular Breathing			p=0.637
Mean (SD)	64.2 (8.04)	65.4 (8.91)	
Min - Max	52 - 79	48 - 89	
Heart Rate, Deep Breathing			p=0.699
Mean (SD)	66.0 (9.16)	67.1 (9.07)	
Min - Max	51 - 82	51 - 91	
HR RB vs DB (p-value)	p=0.006	p=0.094	
Respiratory Rate, Regular Breathing			p=0.013
Mean (SD)	8.4 (3.26)	14.1 (3.26)	
Min - Max	3 - 15	7 - 21	
Respiratory Rate, Deep Breathing			p=0.014
Mean (SD)	4.8 (2.33)	6.9 (3.06)	
Min - Max	2 - 10	3 - 18	
RR RB vs DB (p-value)	p<0.0001	p<0.0001	

Trained years, Mean (SD)			-
Mean (SD)	9.98 (8.98)	-	
Min - Max	1 - 27	-	

* Two-sided Student's t-tests were used to compare NT and T groups. No adjustments were applied for multiple comparisons. Source data are provided as a Source Data file. Abbreviation: DB, deep breathing; HR, heart rate; Max, maximum; Min, minimum, N, number; Nmiss, missing data number; NT, non-trained; RB, regular breathing; SD, standard deviation; T, trained.

Table S2. Summary of Features

Source Data (unit)	Cycle reference	Features computed (unit)	Used in
CSF velocity time course (cm/s)	None	- CSF mean peak velocity (cm/s)	Fig. 1, Fig. 4, Fig. S4, Fig. S6, Fig. S7, Fig. S8
		- CSF mean valley velocity (cm/s)	Fig. S7, Fig. S8
		- CSF pk-to-pk	Fig. S7
		- CSF mean PV (cm/s)	Fig. 2a, Fig. S10
		- CSF mean speed (cm/s)	Fig. 1, Fig. 2b, Fig. 4, Fig. S4, Fig. S5, Fig. S9, Fig. S10, Fig. S11, Fig. S12
CSF flow rate time course (ml/s)	Respiratory	- CSF maximum flow rate (ml/s)	Fig. 2a, Fig. S10
		- CSF minimum flow rate (ml/s)	Fig. S10
CSF volume displacement time course (ml)	Respiratory	- CSF displacement (ml)	Fig. 1, Fig. 2a, Fig. 2b, Fig. 4, Fig. S4, Fig. S5, Fig. S9, Fig. S10, Fig. S11, Fig. S12
		- CSF net flow (µl)	Fig. 1, Fig. 2a, Fig. 2b, Fig. 4, Fig. S4, Fig. S5, Fig. S9, Fig. S10, Fig. S11, Fig. S12
SSS venous flow rate time course (ml/s)	Respiratory	- SSS venous max flow rate (Resp) (ml/s)	Fig. S16

SSS venous volume displacement time course (ml)	Respiratory	- SSS venous displacement (ml)	Fig. 2a, Fig. 3, Fig. 4, Fig. S10, Fig. S12, Fig. S13, Fig. S16
		- SSS venous displacement (Resp) (ml)	Fig. S16
		- SSS venous displacement (Cardio) (ml)	Fig. S16
PPG	Cardiac	- HR (BPM)	Fig. S13, Fig. S16
		- HR displacement (ΔBPM)	Fig. 2a, Fig. 3, Fig. 4, Fig. S10, Fig. S12, Fig. S13, Fig. S16
Respiratory belt	Respiratory	- Respiratory rate (Breaths/min)	Fig. S6, Fig. S11, Fig. S13
		- Breathing length (sec)	Fig. S11
		- Inhale length (sec)	Fig. 2a, Fig. 2b, Fig. 3, Fig. 4, Fig. S10, Fig. S11, Fig. S13, Fig. S16
		- Exhale length (sec)	Fig. S10, Fig. S11, Fig. S13, Fig. S16
		- Inhale:Exhale Ratio	Fig. S11, Fig. S13, Fig. S16
Diaphragm motion MRI	Respiratory	- Lung area displacement	Fig. 2a, Fig. S10, Fig. S11, Fig. S13, Fig. S16
		- Chest displacement	Fig. 2a, Fig. S10, Fig. S11, Fig. S13, Fig. S16
		- Diaphragm displacement	Fig. 2a, Fig. 2b, Fig. 3, Fig. 4, Fig. S10, Fig. S11, Fig. S13, Fig. S16

Abbreviation: CSF, cerebrospinal fluid; Fig, figure; MRI, magnetic resonance imaging; pk-to-pk, peak to peak; PPG, photoplethysmography; PV, peak to valley; SSS, superior sagittal sinus.

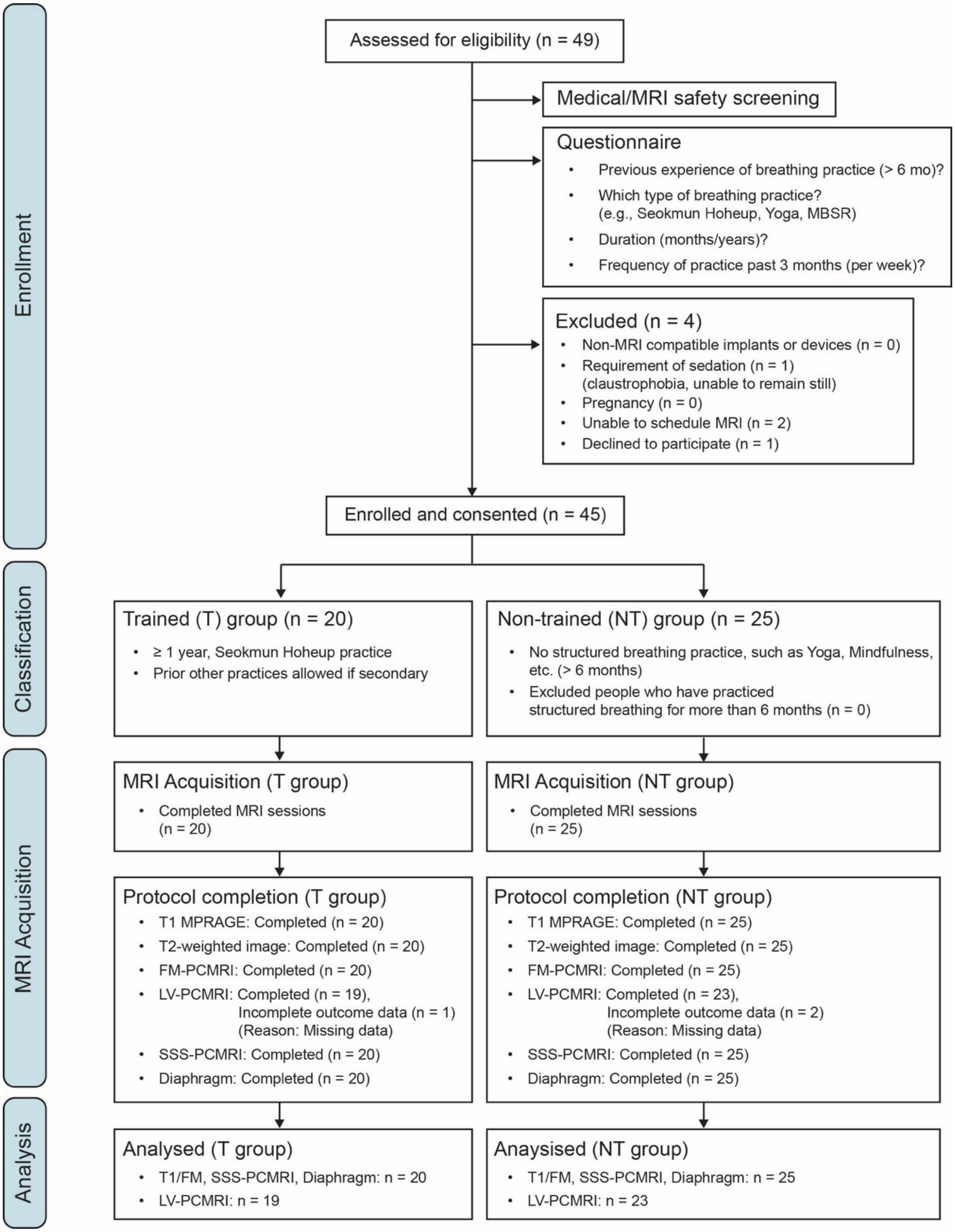

Figure S1. Study flow chart. Flow diagram showing participant screening, exclusions, group allocation (T vs. NT), and final numbers included in each imaging modality (FM-PCMRI, LV-PCMRI, SSS-PCMRI, diaphragm MRI) and corresponding analysis sets. Abbreviation: DB, deep

breathing; FM, foramen magnum; LV, lateral ventricle; MBSR, mindfulness-based stress reduction; MPRAGE, magnetization-prepared rapid gradient-echo; MRI, magnetic resonance imaging; NT, non-trained; PCMRI; phase-contrast MRI; RB, regular breathing; SSS, superior sagittal sinus; T, trained.

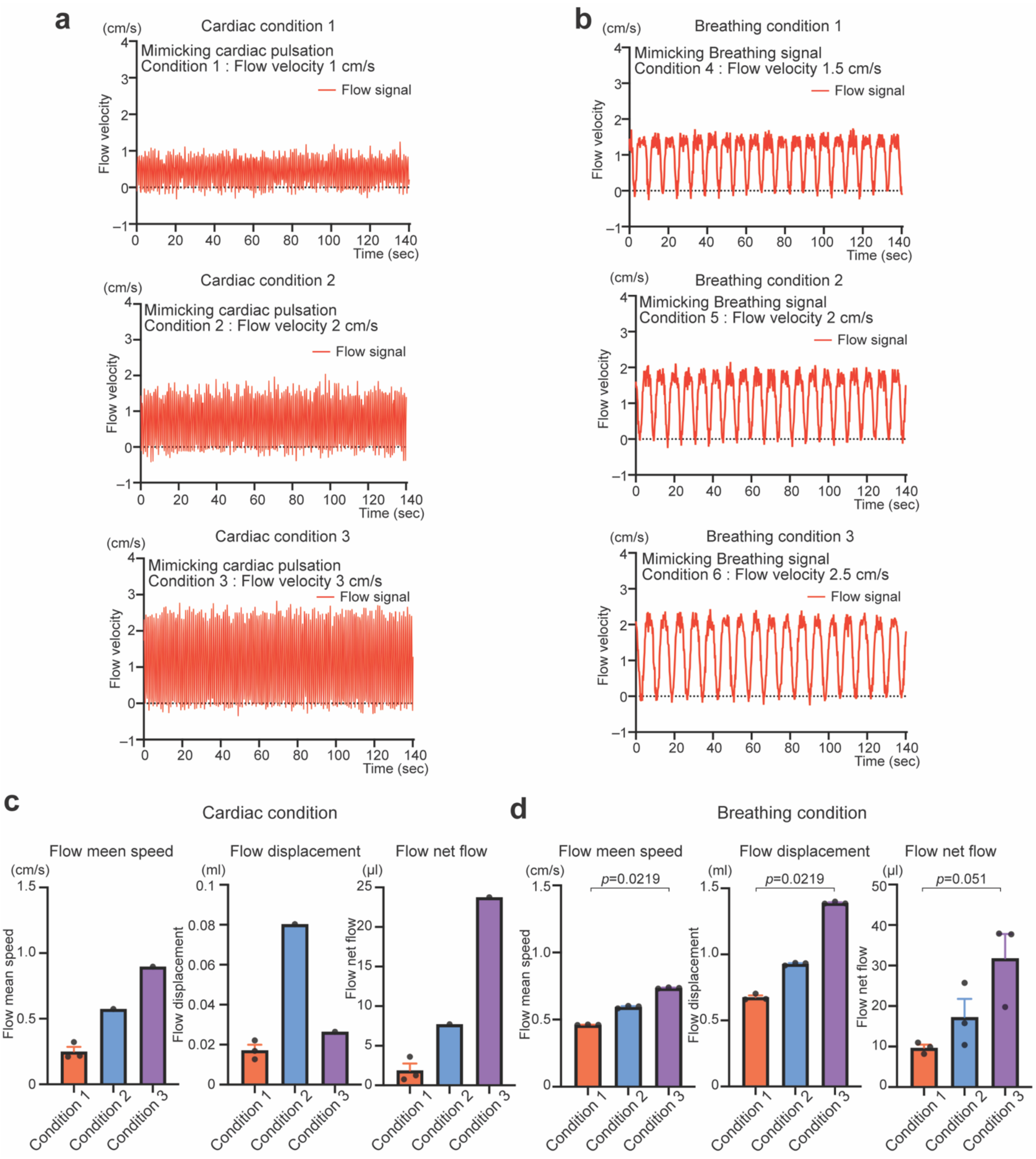

Figure S2. Analysis of PC-MRI signals acquired from the flow phantom. a–b, Extracted velocity signals from a representative scan closely matched the imposed input waveforms under both cardiac-like and respiratory-like oscillatory conditions. c. In the cardiac-like paradigm, net flow increased as flow amplitude rose, whereas displacement did not show a consistent dependence on amplitude. d. In the respiratory-like paradigm, mean speed and displacement

increased significantly with higher flow amplitudes (Kruskal–Wallis test, $H = 7.2$, $p=0.0036$), with the largest difference detected between condition 1 (1.5 cm/s, 8-s cycle) and condition 3 (2.5 cm/s, 8-s cycle; post hoc $p=0.0219$). Net flow also showed a significant overall effect (Kruskal–Wallis test, $H = 5.69$, $p=0.029$), while the post hoc comparison between condition 1 (1.5 cm/s, 8-s cycle) and condition 3 (2.5 cm/s, 8-s cycle) approached significance ($p=0.051$), indicating a trend toward enhanced net flow with increasing respiratory modulation.

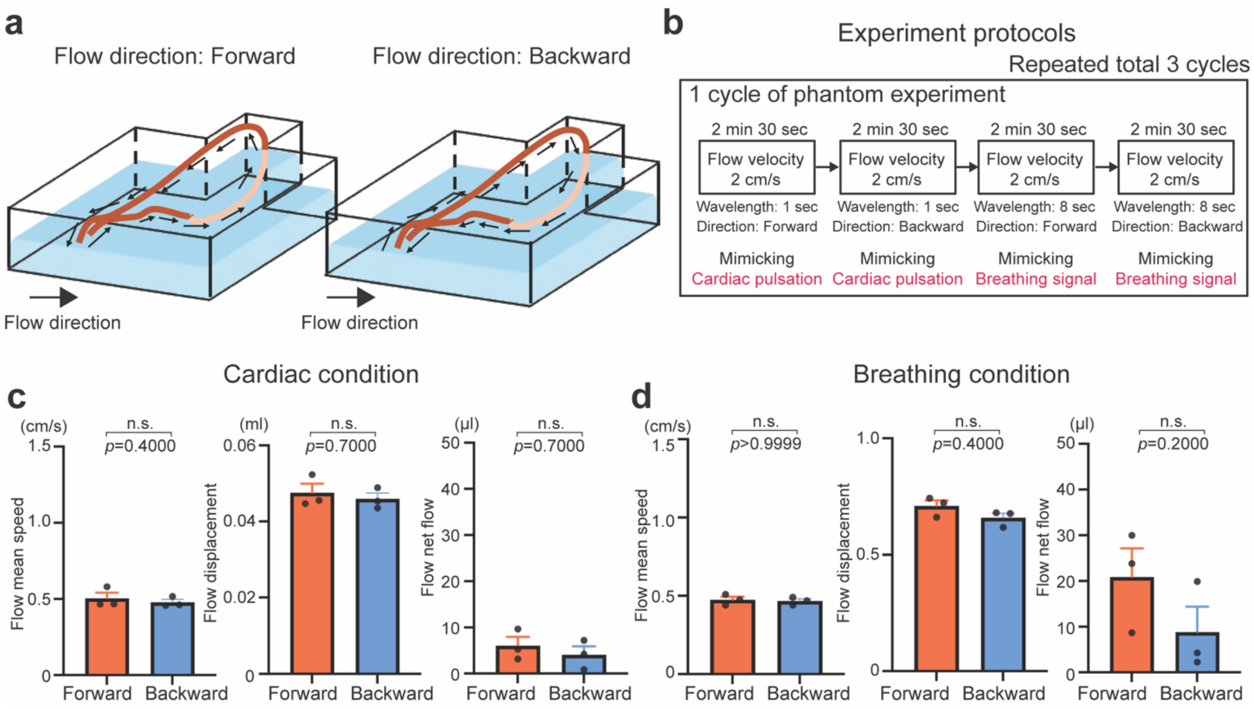

Figure S3. Evaluation of flow directionality using the flow phantom under cardiac-like and respiratory-like conditions. a, Schematic representation of forward and backward flow directions within the phantom. Arrows indicate the flow direction for each condition. b, Experimental protocol to test flow asymmetry. Two-sided Student's t-tests were used to compare forward and backward. Both cardiac-like and respiratory-like waveforms were applied sequentially in forward and backward directions, repeated across three cycles for each condition. c, Under cardiac-like conditions, no statistically significant differences were observed between forward and backward flow for average absolute velocity (p=0.4), displacement (p=0.7), or net flow volume (p=0.7), indicating that direction had no measurable impact under pulsatile cardiac-driven motion. d, Under respiratory-like conditions, no statistically significant directional differences were found for mean speed ($p > 0.9999$), displacement (p=0.4), or net flow volume (p=0.2). However, net flow volume was slightly greater in the forward direction compared to the backward direction, suggesting a potential directional bias in bulk transport under respiratory-driven flow.

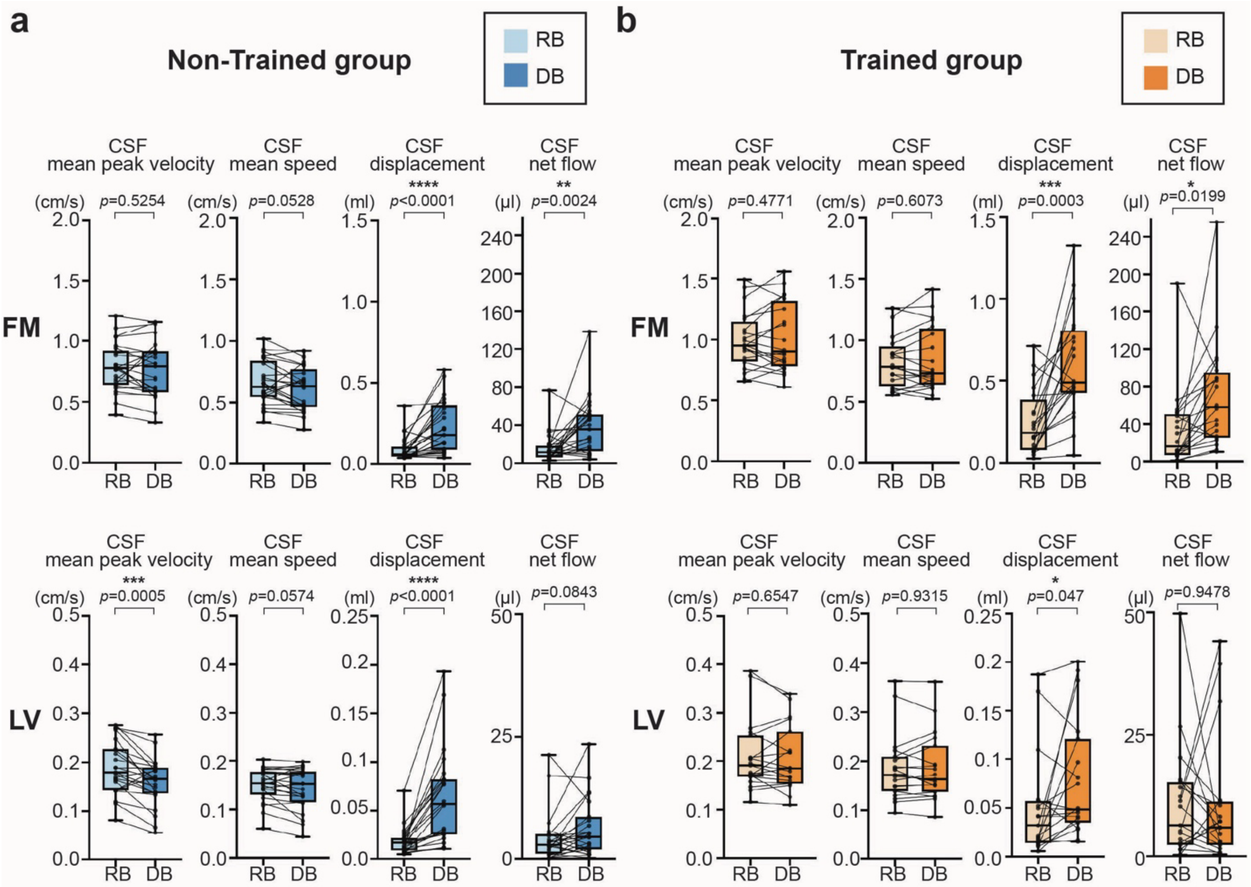

Figure S4. Within-subject comparisons of CSF flow features between RB and DB. a–b, At the FM, CSF displacement increased significantly with DB compared to RB in both T (p=0.0003) and NT (p<0.0001) participants, and CSF net flow also increased with DB in both T (p=0.0199) and NT (p=0.0024). At the LV, CSF displacement increased significantly with DB in both T (p=0.0470) and NT (p<0.0001) participants, whereas CSF net flow did not differ between DB and RB in either group. Statistical comparisons were performed using paired t-tests. No adjustments were applied for multiple comparisons. Source data are provided as a Source Data file. Abbreviation: CSF, cerebrospinal fluid; DB, deep breathing; FM, foramen magnum; LV, lateral ventricle; NT, non-trained; RB, regular breathing; T, trained.

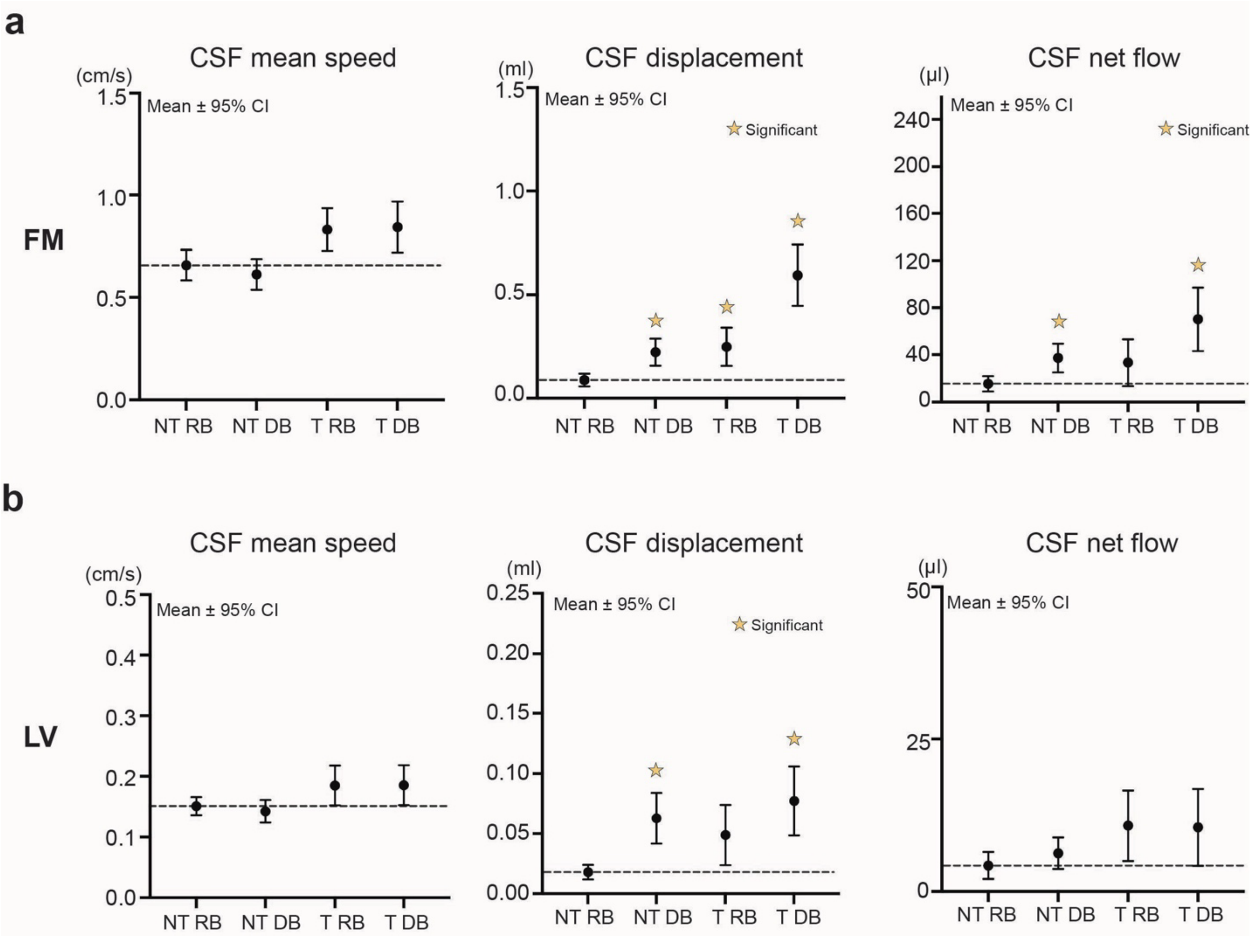

Figure S5. Confidence interval–based comparisons of CSF flow features across groups and breathing conditions. Confidence interval (mean ± 95% CI) analyses were performed using NT-RB as the reference group. a, FM. b, LV. CSF flow features include CSF mean speed, CSF displacement, and CSF net flow. Stars indicate statistical significance. Source data are provided as a Source Data file. Abbreviation: CI, confidence interval; CSF, cerebrospinal fluid; DB, deep breathing; FM, foramen magnum; LV, lateral ventricle; NT, non-trained; RB, regular breathing; T, trained.

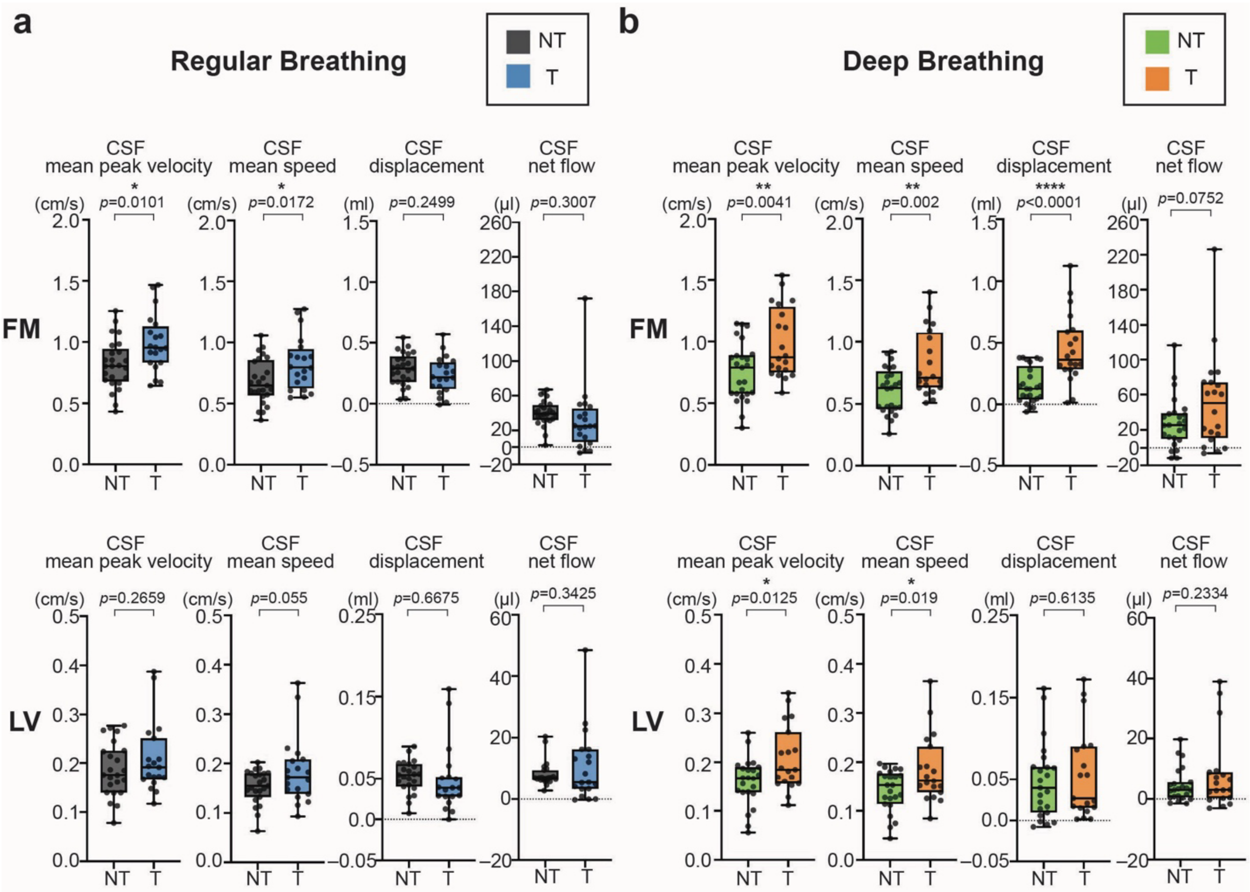

Figure S6. Sensitivity analysis controlling for respiratory rate (RR). A sensitivity analysis was conducted by regressing out RR to assess the influence of breathing frequency on CSF flow measures. Two-sided Student's t-tests were used to compare NT and T groups. No adjustments were applied for multiple comparisons. a, RB: CSF mean peak velocity and CSF mean speed remained unchanged after adjustment. At the FM, group differences in CSF displacement and CSF net flow were no longer significant. At the LV, group differences in CSF displacement and CSF net flow were no longer significant. b, DB: the difference between groups persisted at the FM. Source data are provided as a Source Data file. Abbreviation: CSF, cerebrospinal fluid; DB, deep breathing; FM, foramen magnum; LV, lateral ventricle; NT, non-trained; RB, regular breathing; RR, respiratory rate; T, trained.

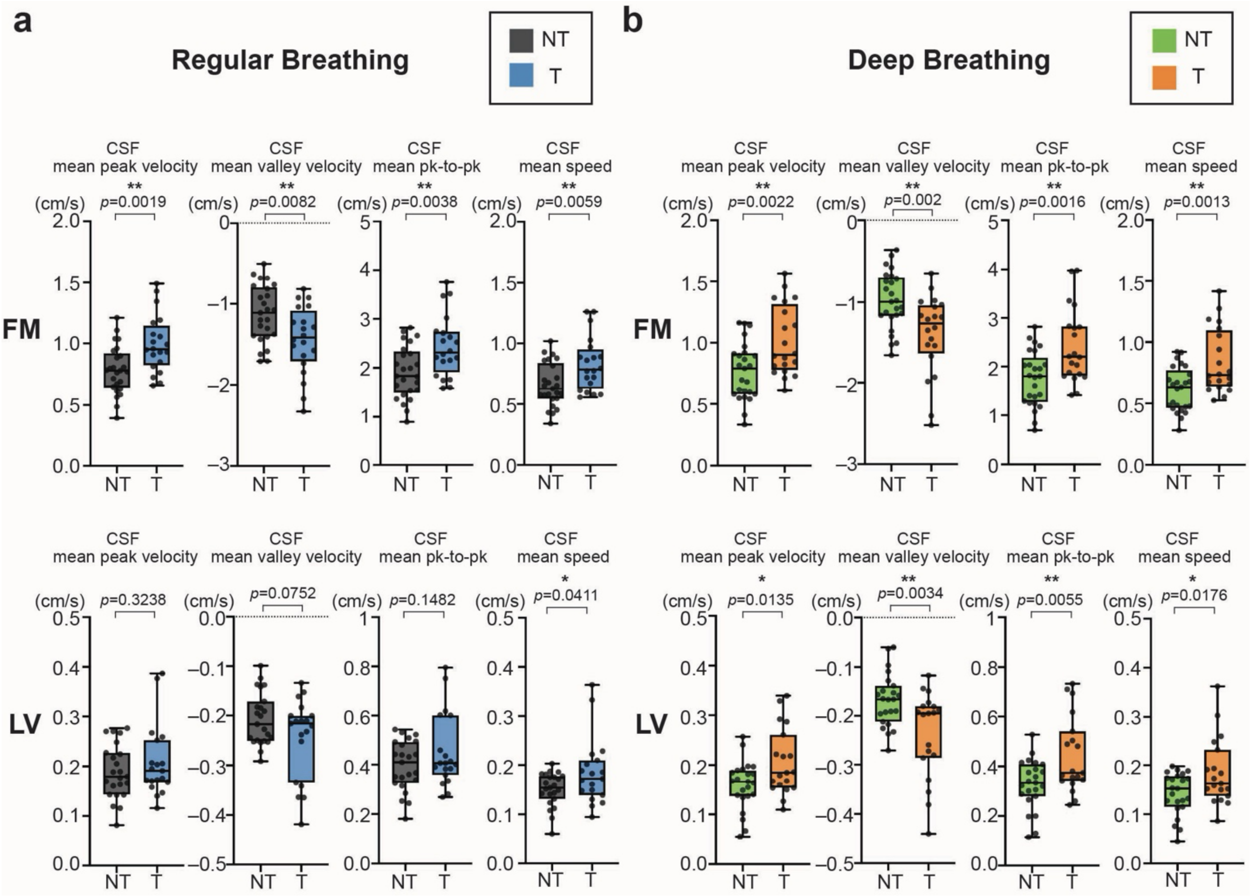

Figure S7. Group comparisons of peak-based CSF velocity features. Representative peak-based velocity features are shown for RB and DB conditions. Two-sided Student's t-tests were used to compare NT and T groups. No adjustments were applied for multiple comparisons. a, During RB, the FM showed group differences across all CSF velocity measures, including CSF mean peak velocity, CSF mean valley velocity, CSF mean peak-to-peak velocity, and CSF mean speed. At the LV, only CSF mean speed differed between NT and T. b, During DB, all peak-based velocity features showed significant differences between NT and T at both the FM and LV. Source data are provided as a Source Data file. Abbreviation: CSF, cerebrospinal fluid; DB, deep breathing; FM, foramen magnum; LV, lateral ventricle; NT, non-trained; pk-to-pk, peak to peak; RB, regular breathing; T, trained.

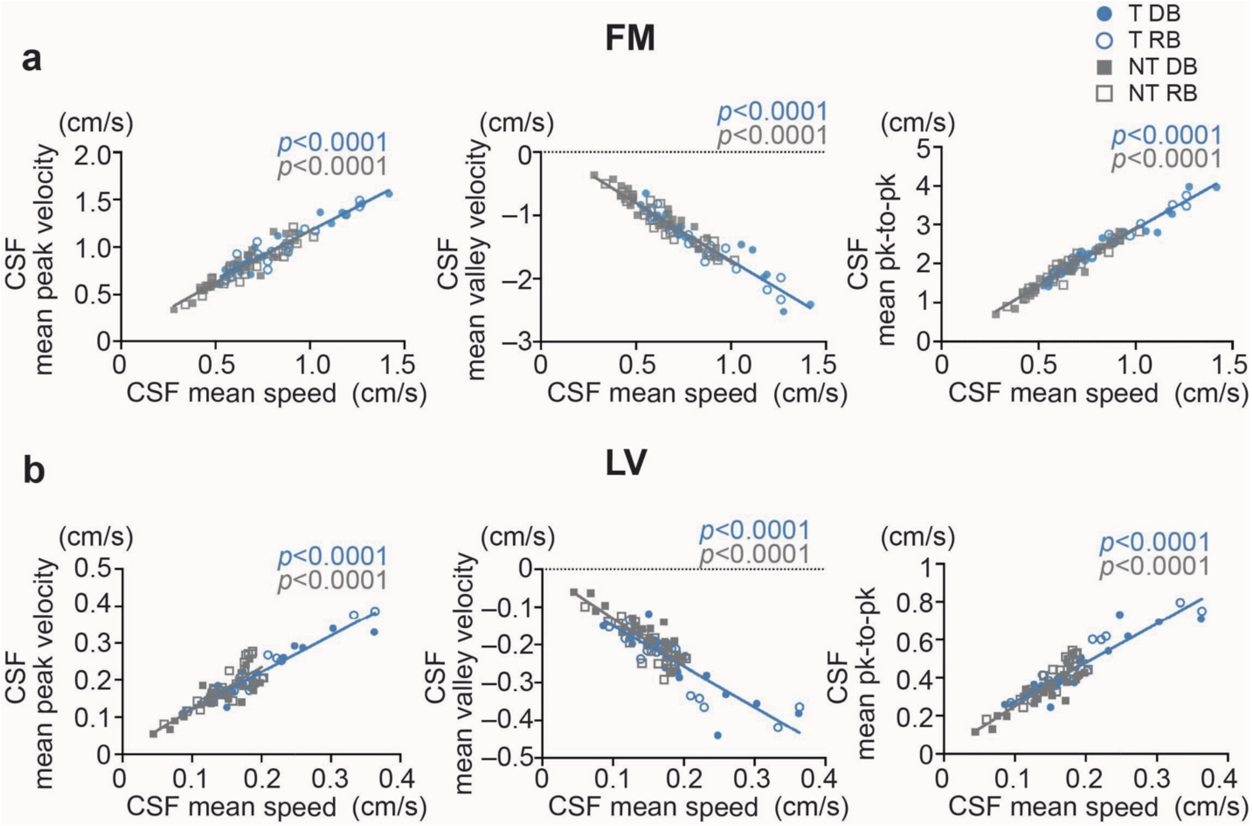

Figure S8. Correlations among peak-based CSF velocity features. All peak-based velocity features showed very high correlations with one another ($p < 0.0001$). Linear regression analyses were performed using Pearson's correlation with two-sided tests. Pearson's correlation analyses were conducted separately in T (light blue) and NT (gray). a, FM: correlations between CSF mean peak velocity and CSF mean speed, CSF mean valley velocity and CSF mean speed, and CSF mean peak-to-peak velocity and CSF mean speed all showed $p < 0.0001$. b, LV: correlations between CSF mean peak velocity and CSF mean speed, CSF mean valley velocity and CSF mean speed, and CSF mean peak-to-peak velocity and CSF mean speed all showed $p < 0.0001$. Source data are provided as a Source Data file. Abbreviation: CSF, cerebrospinal fluid; DB, deep breathing; FM, foramen magnum; LV, lateral ventricle; NT, non-trained; pk-to-pk, peak to peak; RB, regular breathing; T, trained.

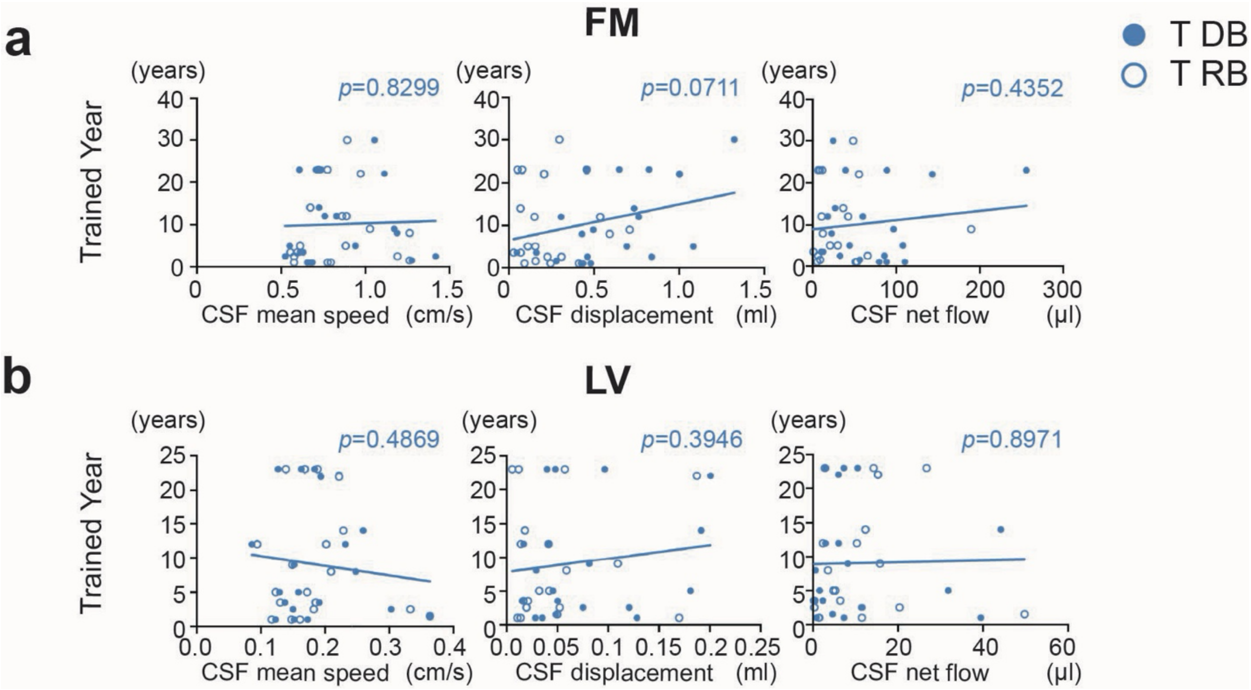

Figure S9. Correlations between years of training and CSF flow features. Linear regression analyses were performed using Pearson's correlation with two-sided tests. Pearson's correlation analyses were performed to examine the relationship between years of training and CSF flow features, including CSF mean speed, CSF displacement, and CSF net flow in T group. a. Correlation analysis was performed on the full dataset, while data points are displayed separately for RB (T-RB, light blue open circles) and DB (T-DB, light blue filled circles). No significance was shown with Pearson's correlation between trained year and CSF features in the FM. b. Correlation analysis was performed showing no significance between trained year and CSF features in the LV. Source data are provided as a Source Data file. Abbreviation: CSF, cerebrospinal fluid; DB, deep breathing; FM, foramen magnum; LV, lateral ventricle; RB, regular breathing; T, trained.

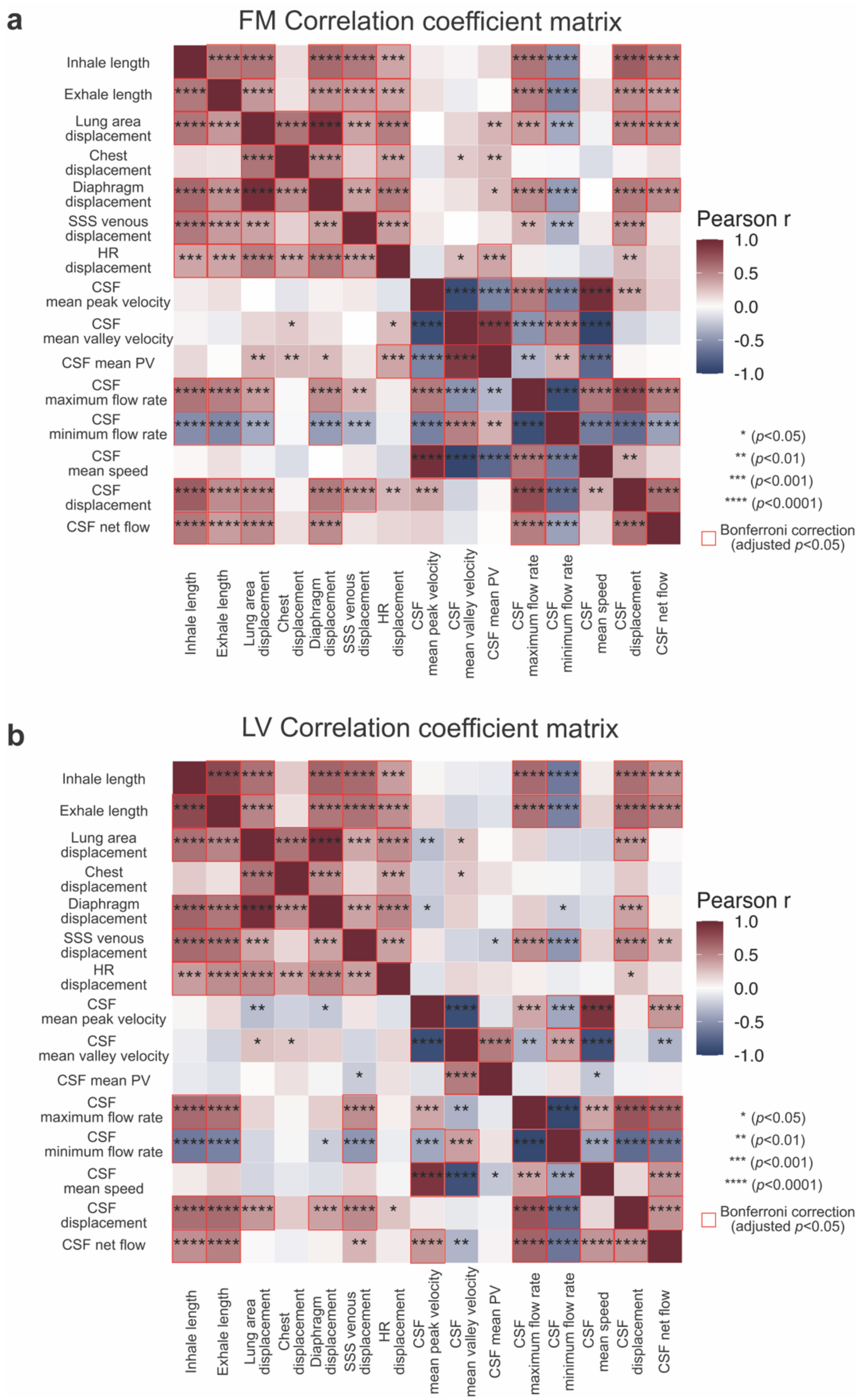

Figure S10. Correlation coefficient matrices of respiratory, physiological, and CSF flow features. a, FM correlation matrix. b, LV correlation matrix. Pearson's correlation coefficients (r)

are shown for the following features: Inhale Length, Exhale Length, Lung Area displacement, Chest displacement, Diaphragm displacement, SSS Venous displacement, HR displacement, CSF Mean Peak, CSF Mean Valley, CSF MeanPV (mean of peak and valley velocity), CSF MaxFlow rate, CSF MinFlow, CSF mean speed, CSF displacement, and CSF Net Flow. Color scale represents Pearson's r values from –1.0 (blue) to +1.0 (red). Significance levels are indicated as: *p<0.05, **p<0.01, ***p<0.001, ****p<0.0001. Correlations that survived Bonferroni correction ($p < 0.05$) are marked with red boxes. Source data are provided as a Source Data file. Abbreviation: CSF, cerebrospinal fluid; DB; deep breathing; FM, foramen magnum; HR, heart rate; LV, lateral ventricle; PV, peak to valley; SSS, superior sagittal sinus.

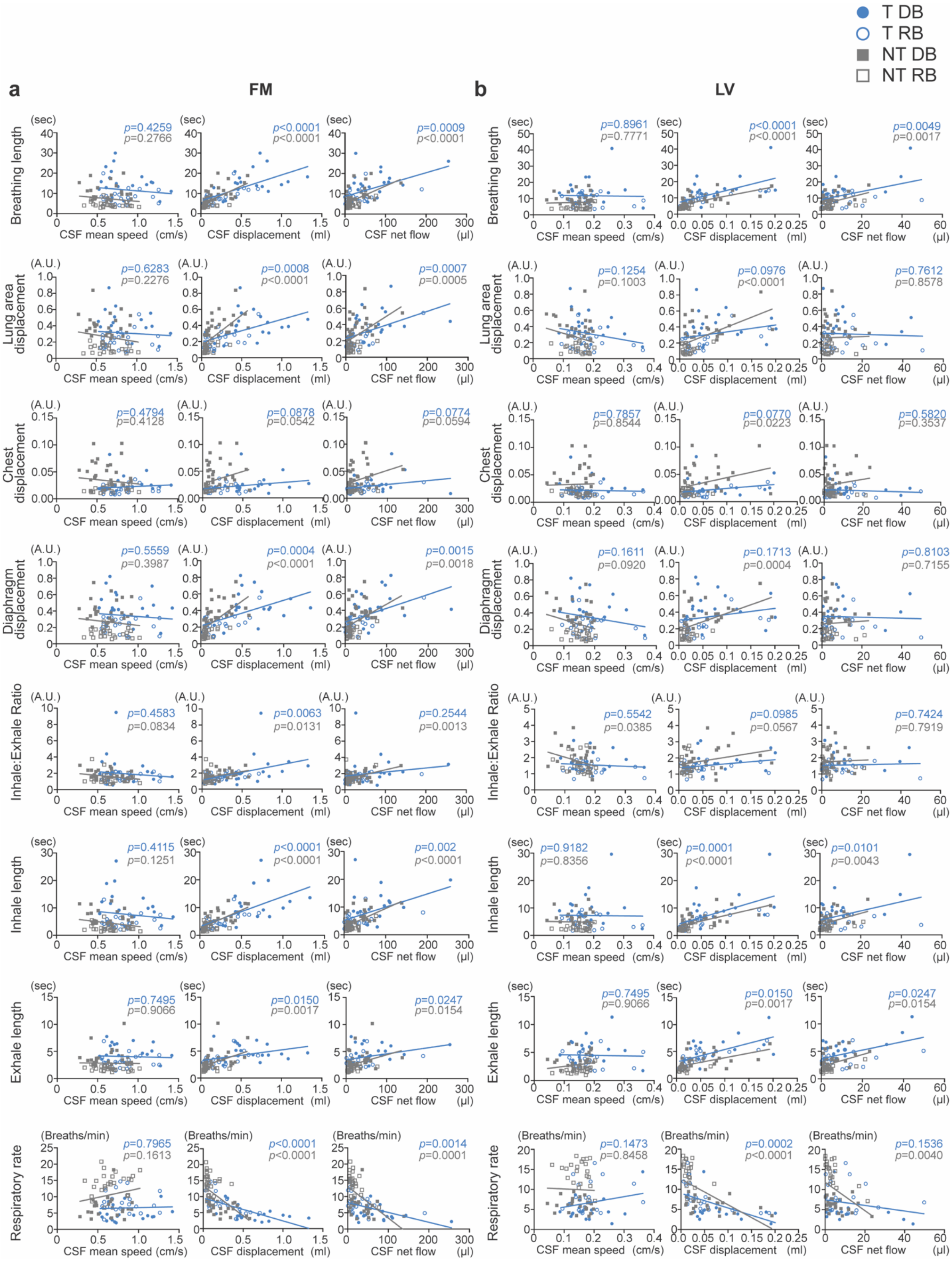

Figure S11. Correlation matrices of respiratory and CSF flow features. Linear regression

analyses were performed using Pearson's correlation with two-sided tests. Pearson's correlation analyses were conducted separately in T (light blue) and NT (gray). a, Correlation matrix for features measured at the FM. b, Correlation matrix for features measured at the LV. Respiratory features include breathing length, lung area displacement, chest displacement, diaphragm displacement, inhale-to-exhale ratio, inhale length, exhale length, and respiratory rate. CSF flow features include CSF mean speed, CSF displacement, and CSF net flow. Source data are provided as a Source Data file. Abbreviation: A.U., arbitrary unit; CSF, cerebrospinal fluid; DB, deep breathing; FM, foramen magnum; LV, lateral ventricle; NT, non-trained; RB, regular breathing; T, trained.

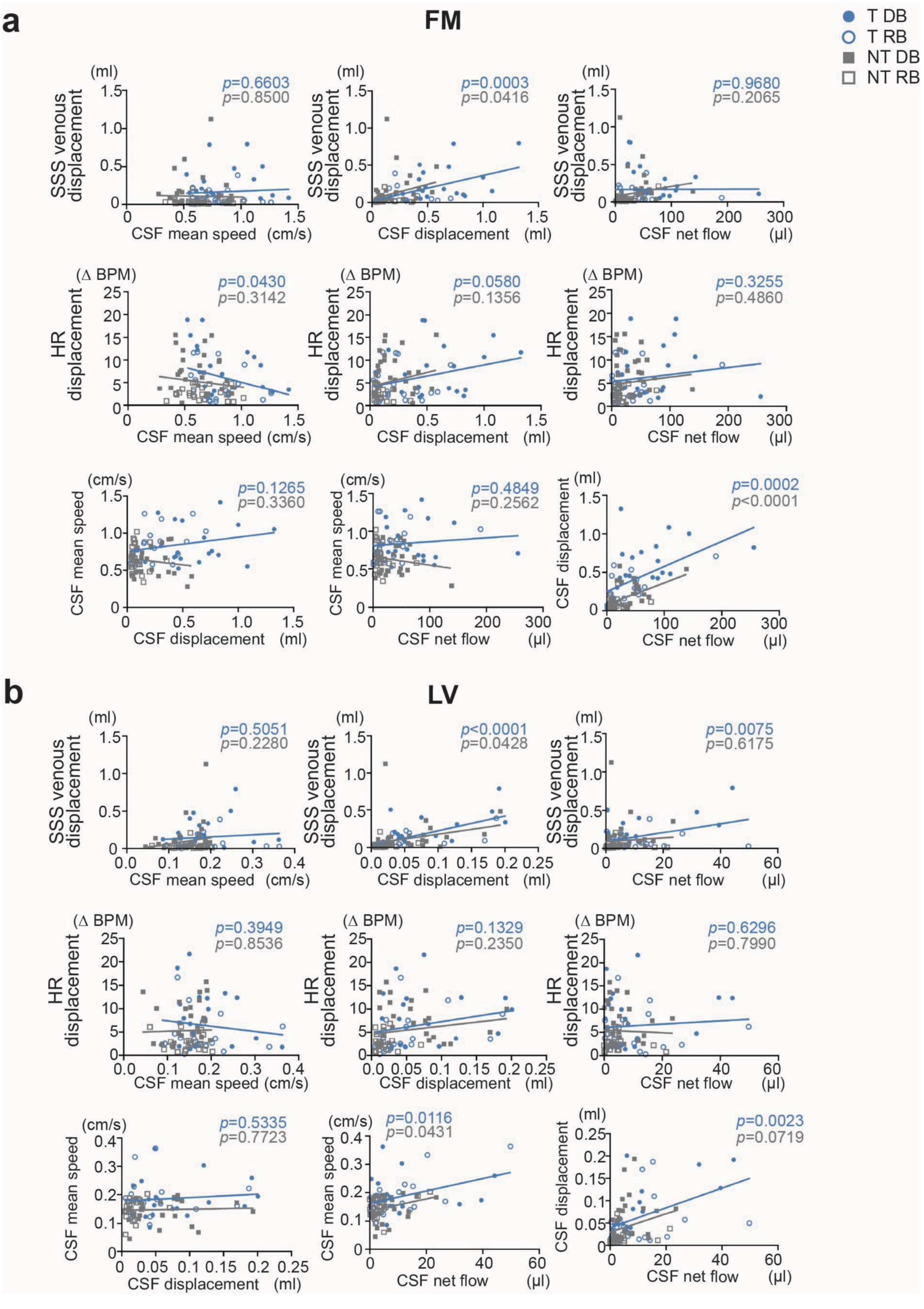

a
FM
b
LV
T DB
T RB
NT DB
NT RB
SSS venous displacement (ml)
HR displacement (Δ BPM)
CSF mean speed (cm/s)
CSF displacement (ml)
CSF net flow (μl)
p=0.6603
p=0.8500
p=0.0003
p=0.0416
p=0.9680
p=0.2065
p=0.0430
p=0.3142
p=0.0580
p=0.1356
p=0.3255
p=0.4860
p=0.1265
p=0.3360
p=0.4849
p=0.2562
p=0.0002
p<0.0001
p=0.5051
p=0.2280
p<0.0001
p=0.0428
p=0.0075
p=0.6175
p=0.3949
p=0.8536
p=0.1329
p=0.2350
p=0.6296
p=0.7990
p=0.5335
p=0.7723
p=0.0116
p=0.0431
p=0.0023
p=0.0719

Figure S12. Correlations of physiological features with CSF flow features, and correlations among CSF flow features. a, Correlation matrix for the FM. b, Correlation matrix for the LV. Physiological features included SSS displacement and HR displacement. CSF flow features included CSF mean speed, CSF displacement, and CSF net flow. Linear regression analyses were performed using Pearson's correlation with two-sided tests. Pearson's correlation analyses were conducted separately in T (light blue) and NT (gray). Source data are provided as a Source Data file. Abbreviation: BPM, beats per minute; CSF, cerebrospinal fluid; DB, deep breathing; FM, foramen magnum; HR, heart rate; LV, lateral ventricle; NT, non-trained; RB, regular breathing; SSS, superior sagittal sinus; T, trained.

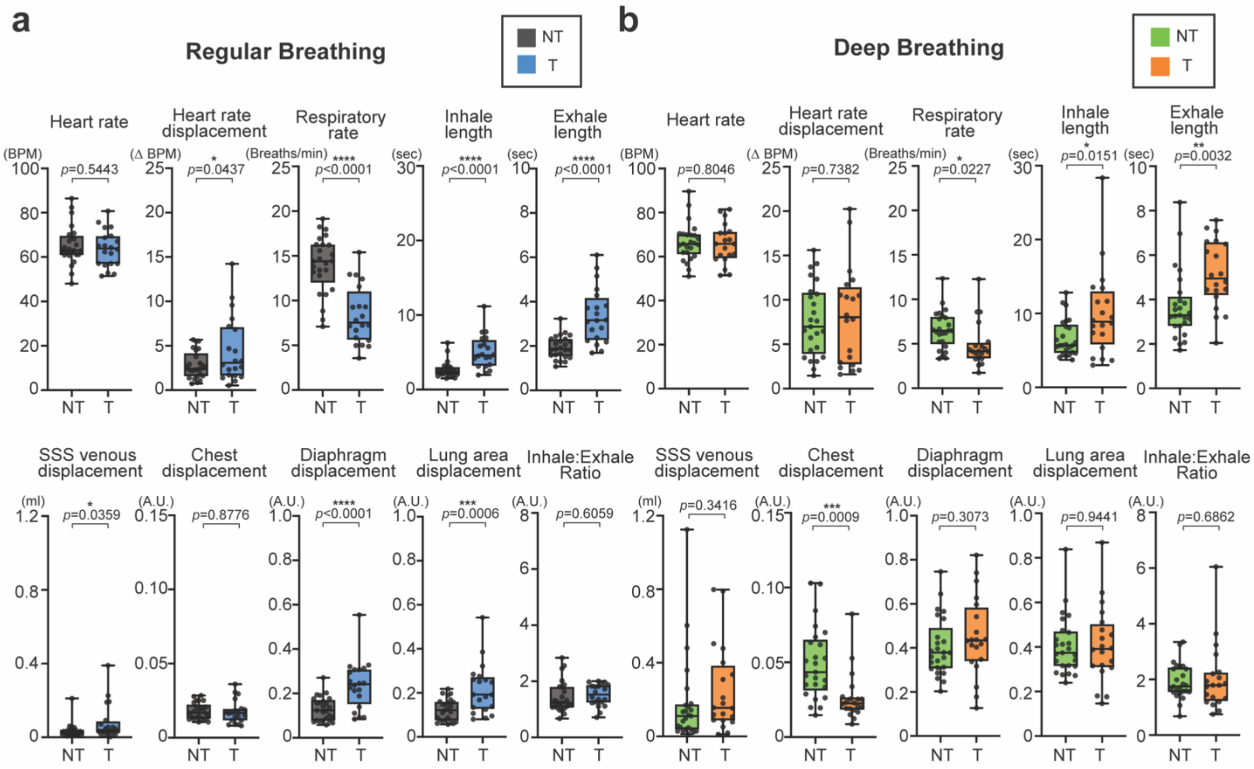

Figure S13. Comparisons of respiratory and physiological features between T and NT groups. Two-sided Student's t-tests were used to compare NT and T groups. a, RB. b, DB. Features compared include HR, HR displacement, respiratory rate, inhale length, exhale length, SSS venous displacement, chest displacement, diaphragm displacement, lung area displacement, and inhale-to-exhale ratio. In the whisker plots, NT during RB is shown in gray, T during RB in light blue, NT during DB in light green, and T during DB in orange for comparison. Statistical significance is indicated as p<0.05 (*), p<0.01 (**), p<0.001 (***), and p<0.0001 (****). No adjustments were applied for multiple comparisons. Source data are provided as a Source Data file. Abbreviation: BPM, beats per minute; DB, deep breathing; HR, heart rate; NT, non-trained; RB, regular breathing; SSS, superior sagittal sinus; T, trained.

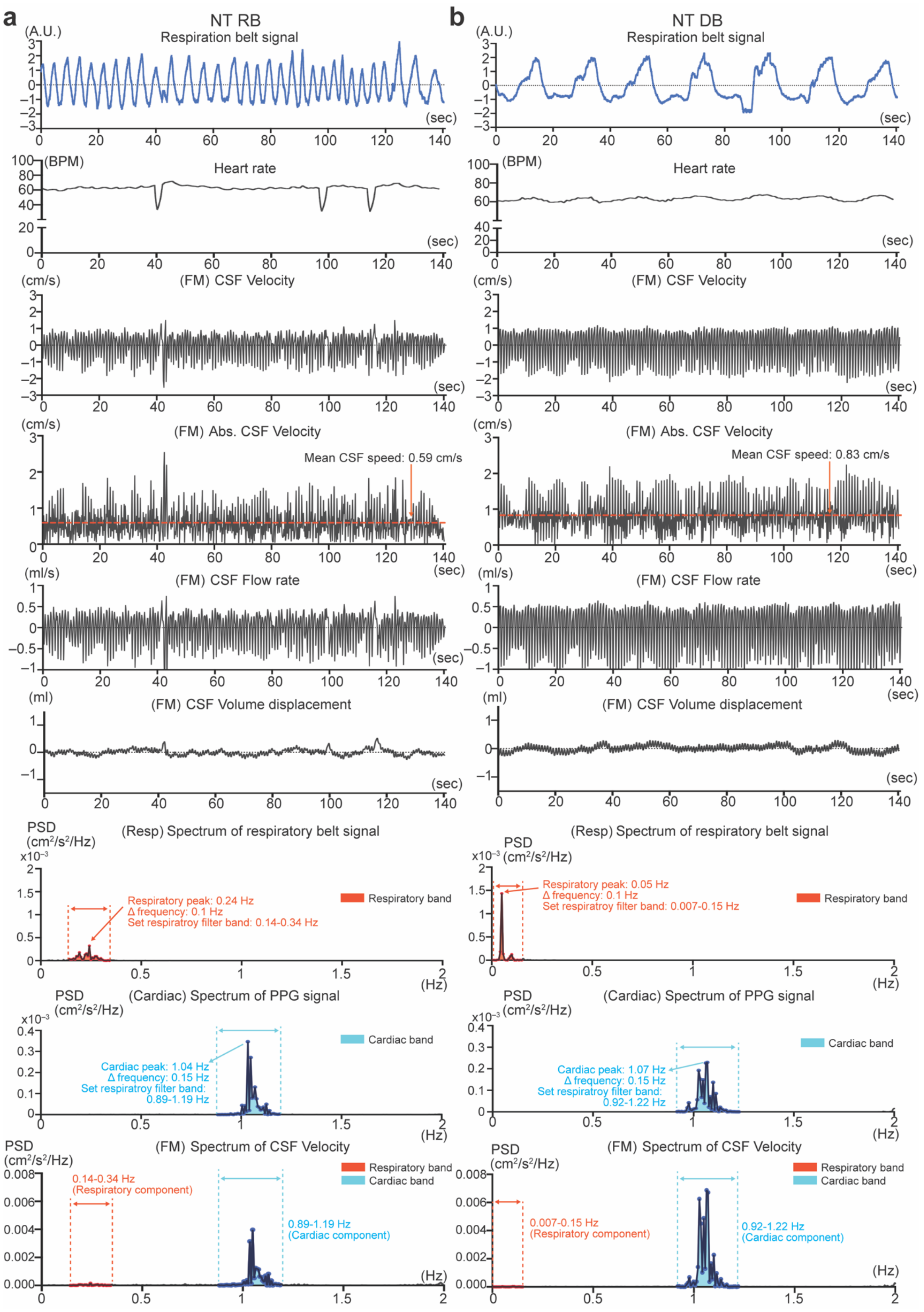

a
NT RB
Respiration belt signal
Heart rate
(FM) CSF Velocity
(FM) Abs. CSF Velocity
Mean CSF speed: 0.59 cm/s
(FM) CSF Flow rate
(FM) CSF Volume displacement
(Resp) Spectrum of respiratory belt signal
Respiratory peak: 0.24 Hz
Δ frequency: 0.1 Hz
Set respiratroy filter band: 0.14-0.34 Hz
Respiratory band
(Cardiac) Spectrum of PPG signal
Cardiac peak: 1.04 Hz
Δ frequency: 0.15 Hz
Set respiratroy filter band: 0.89-1.19 Hz
Cardiac band
(FM) Spectrum of CSF Velocity
0.14-0.34 Hz
(Respiratory component)
0.89-1.19 Hz
(Cardiac component)
b
NT DB
Respiration belt signal
Heart rate
(FM) CSF Velocity
(FM) Abs. CSF Velocity
Mean CSF speed: 0.83 cm/s
(FM) CSF Flow rate
(FM) CSF Volume displacement
(Resp) Spectrum of respiratory belt signal
Respiratory peak: 0.05 Hz
Δ frequency: 0.1 Hz
Set respiratroy filter band: 0.007-0.15 Hz
Respiratory band
(Cardiac) Spectrum of PPG signal
Cardiac peak: 1.07 Hz
Δ frequency: 0.15 Hz
Set respiratroy filter band: 0.92-1.22 Hz
Cardiac band
(FM) Spectrum of CSF Velocity
0.007-0.15 Hz
(Respiratory component)
0.92-1.22 Hz
(Cardiac component)

Figure S14. Representative case from a NT participant. Each row displays the respiratory belt signal, HR, CSF velocity (cm/s), absolute CSF velocity (cm/s), CSF flow rate (ml/s), and CSF volume displacement (ml). Panels a and b correspond to RB and DB conditions, respectively. Frequency-domain spectral analyses of the respiratory belt, PPG, and CSF velocity time series are shown alongside, illustrating the relative power of respiratory and cardiac components under each condition. The participant (male, age 62) belonged to the upper 50% group with high FM net flow. Under RB, CSF mean speed, displacement, and net flow were 0.59 cm/s, 0.10 ml, and 20.27 μl, respectively. During DB, these values changed to 0.83 cm/s, 0.21 ml, and 64.72 μl. Abbreviation: A.U., arbitrary unit; BPM, beats per minute; CSF, cerebrospinal fluid; DB, deep breathing; FM, foramen magnum; HR, heart rate; NT, non-trained; PPG, photoplethysmography; PSD, power spectral density; RB, regular breathing; T, trained.

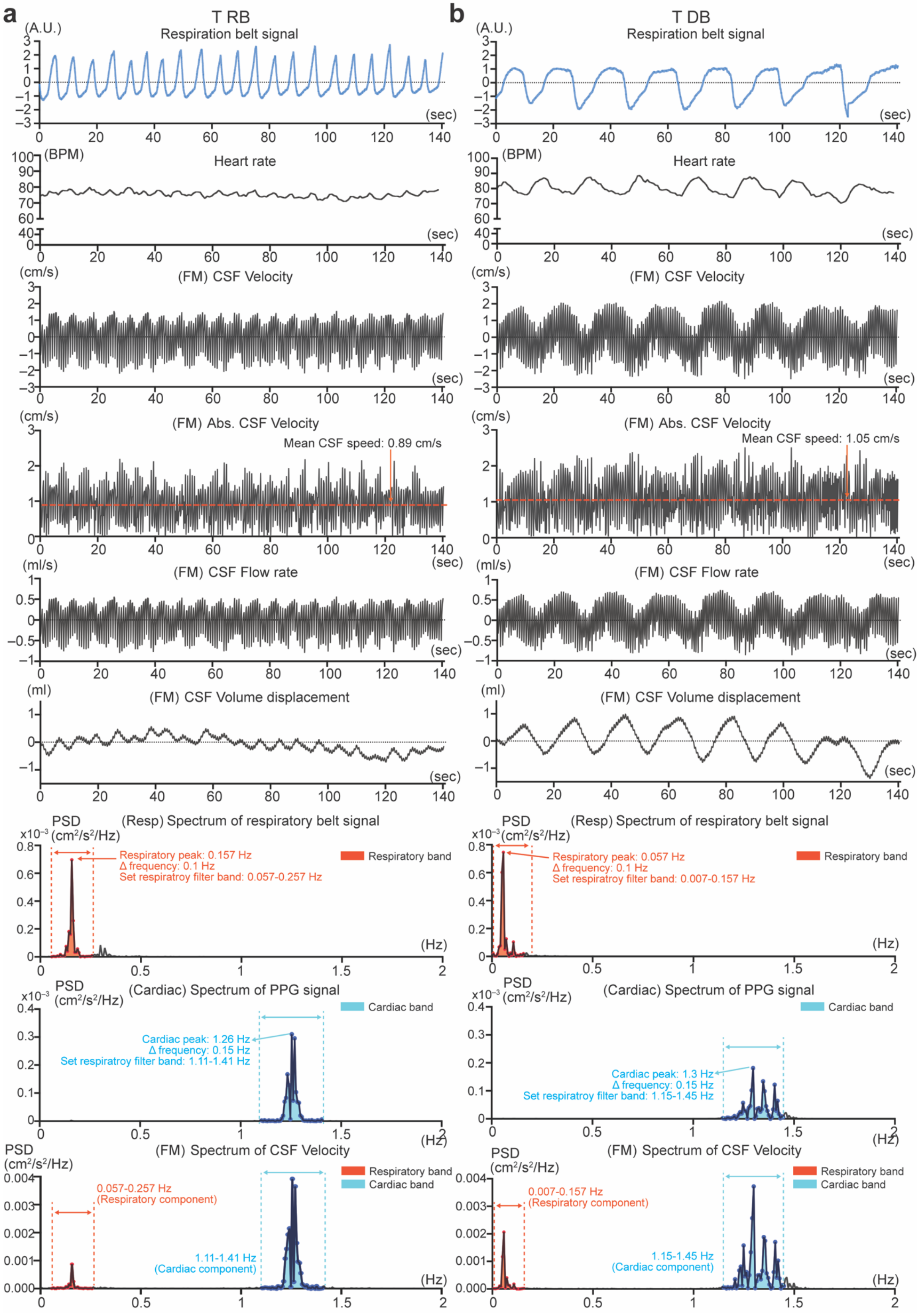

a
T RB
Respiration belt signal
Heart rate
(FM) CSF Velocity
(FM) Abs. CSF Velocity
Mean CSF speed: 0.89 cm/s
(FM) CSF Flow rate
(FM) CSF Volume displacement
(Resp) Spectrum of respiratory belt signal
Respiratory peak: 0.157 Hz
Δ frequency: 0.1 Hz
Set respiratroy filter band: 0.057-0.257 Hz
Respiratory band
(Cardiac) Spectrum of PPG signal
Cardiac peak: 1.26 Hz
Δ frequency: 0.15 Hz
Set respiratroy filter band: 1.11-1.41 Hz
Cardiac band
(FM) Spectrum of CSF Velocity
0.057-0.257 Hz
(Respiratory component)
1.11-1.41 Hz
(Cardiac component)
b
T DB
Respiration belt signal
Heart rate
(FM) CSF Velocity
(FM) Abs. CSF Velocity
Mean CSF speed: 1.05 cm/s
(FM) CSF Flow rate
(FM) CSF Volume displacement
(Resp) Spectrum of respiratory belt signal
Respiratory peak: 0.057 Hz
Δ frequency: 0.1 Hz
Set respiratroy filter band: 0.007-0.157 Hz
(Cardiac) Spectrum of PPG signal
Cardiac peak: 1.3 Hz
Δ frequency: 0.15 Hz
Set respiratroy filter band: 1.15-1.45 Hz
(FM) Spectrum of CSF Velocity
0.007-0.157 Hz
(Respiratory component)
1.15-1.45 Hz
(Cardiac component)

Figure S15. Representative case from a T participant. Each row displays the respiratory belt signal, HR, CSF velocity (cm/s), absolute CSF velocity (cm/s), CSF flow rate (mL/s), and CSF volume displacement (ml). Panels a and b correspond to RB and DB conditions, respectively. Frequency-domain spectral analyses of the respiratory belt, PPG, and CSF velocity time series are shown alongside, illustrating the relative power of respiratory and cardiac components under each condition. The participant (male, age 59) belonged to the upper 50% group with high FM net flow. Under RB, CSF mean speed, displacement, and net flow were 0.89 cm/s, 0.30 mL, and 48.59 µl, respectively. During DB, these values changed to 1.05 cm/s, 1.32 mL, and 24.64 µl. Abbreviation: A.U., arbitrary unit; BPM, beats per minute; CSF, cerebrospinal fluid; DB, deep breathing; FM, foramen magnum; HR, heart rate; NT, non-trained; PPG, photoplethysmography; PSD, power spectral density; RB, regular breathing; T, trained.

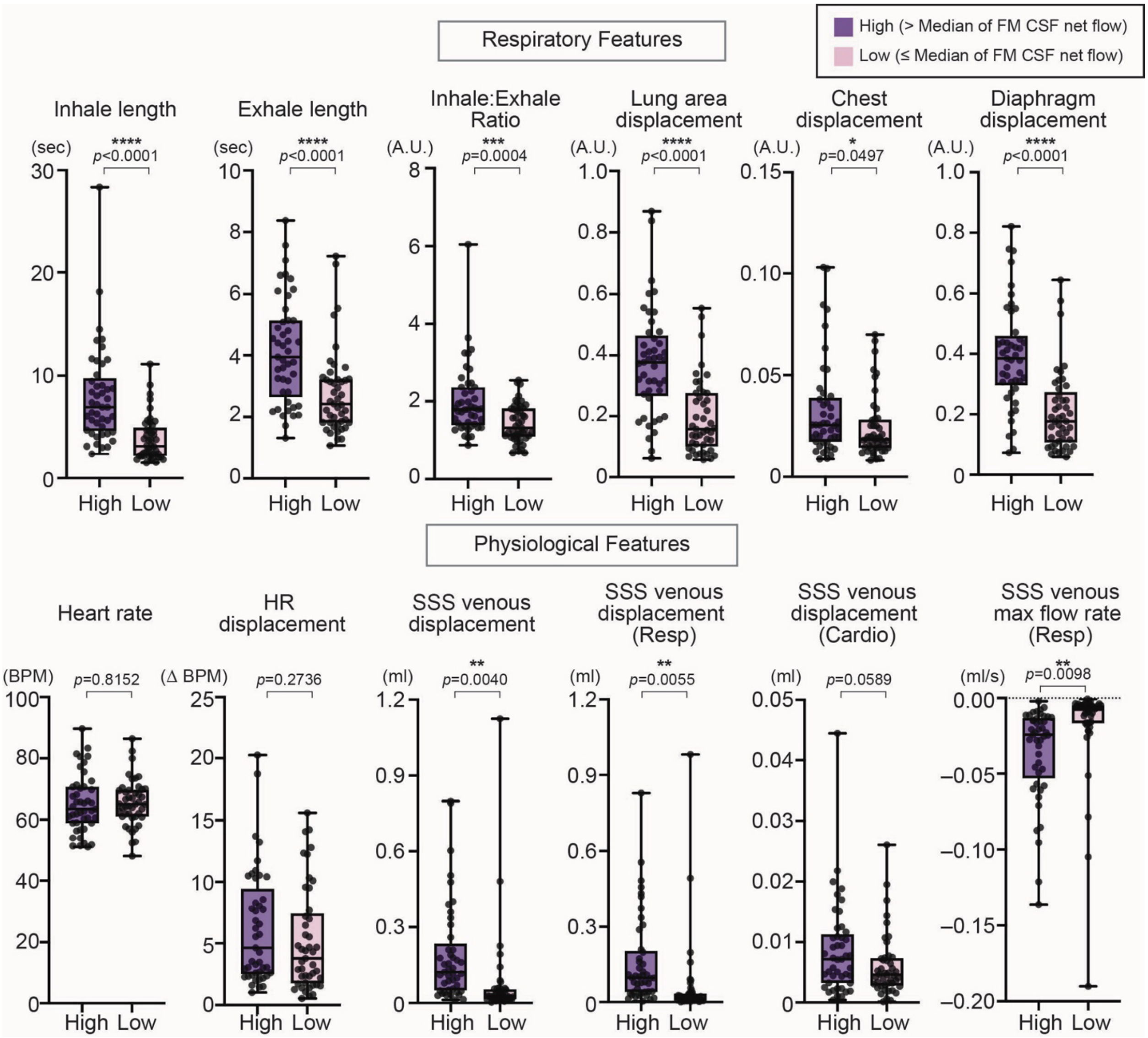

Figure S16. Respiratory and physiological features linked to higher CSF net flow across participants. Participants were divided into High and Low groups based on the median of the FM CSF net flow. Group differences were assessed using two-sided Student's t-tests. Participant numbers (T = 19, NT = 23) reflect those who had both FM and LV data. Features include inhale length, exhale length, inhale-to-exhale ratio, lung area displacement, chest displacement, diaphragm displacement, HR, HR displacement, SSS venous displacement, SSS venous displacement (respiratory component), SSS venous displacement (cardiac component), and SSS venous maximum flow rate (respiratory component). Data are shown as whisker plots, with the High group in purple and the Low in pink. Statistical significance is indicated as *p<0.05, **p<0.01, ***p<0.001, and ****p<0.0001. No adjustments were applied for multiple

comparisons. Source data are provided as a Source Data file. Abbreviation: A.U., arbitrary unit; BPM, beats per minute; CSF, cerebrospinal fluid; FM, foramen magnum; HR, heart rate; NT, non-trained; SSS, superior sagittal sinus; T, trained.

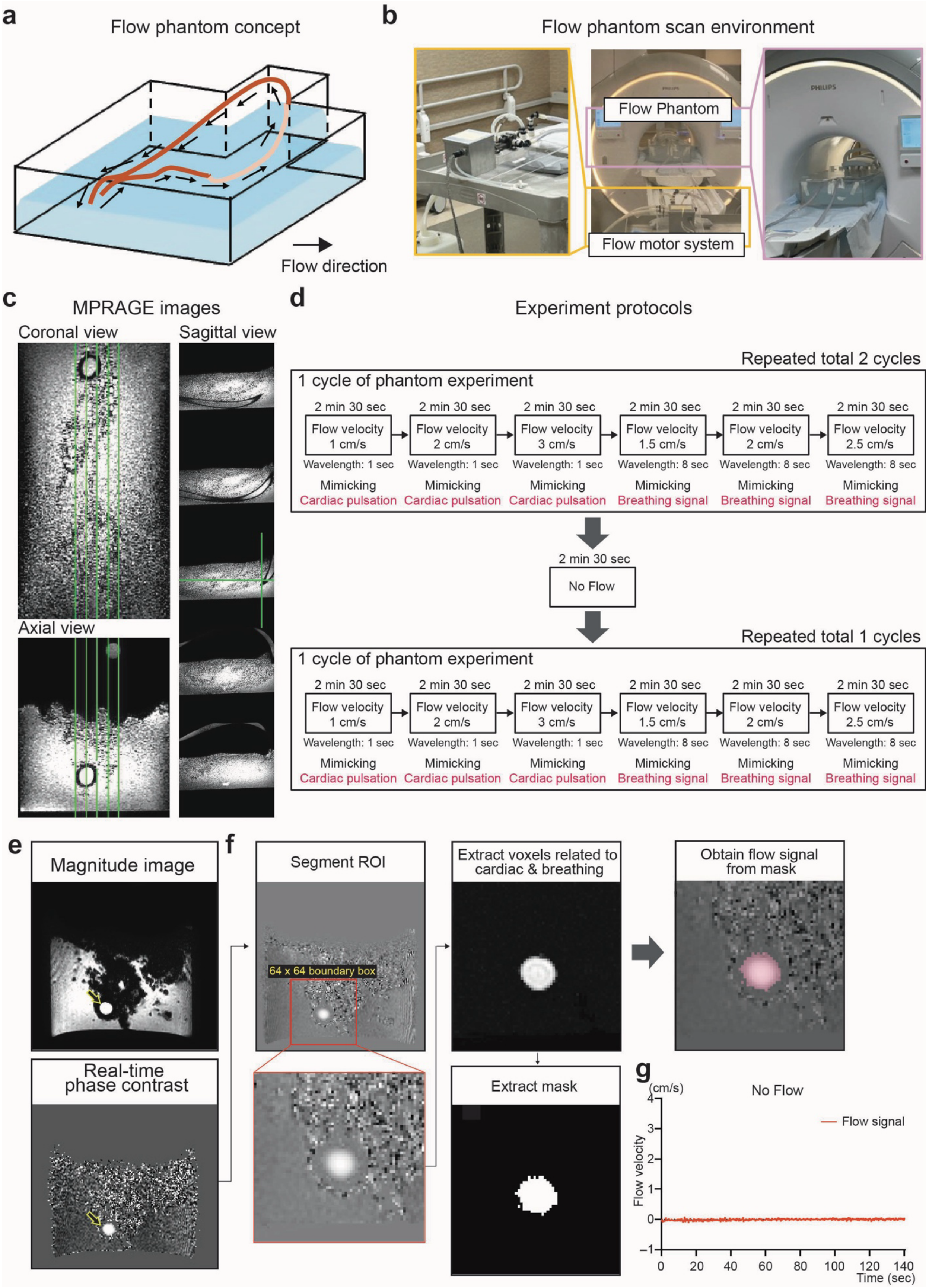
a
Flow phantom concept
Flow direction
b
Flow phantom scan environment
Flow Phantom
Flow motor system
PHILIPS
c
MPRAGE images
Coronal view
Sagittal view
Axial view
d
Experiment protocols
Repeated total 2 cycles
1 cycle of phantom experiment
2 min 30 sec
Flow velocity 1 cm/s
Wavelength: 1 sec
Mimicking Cardiac pulsation
2 min 30 sec
Flow velocity 2 cm/s
Wavelength: 1 sec
Mimicking Cardiac pulsation
2 min 30 sec
Flow velocity 3 cm/s
Wavelength: 1 sec
Mimicking Cardiac pulsation
2 min 30 sec
Flow velocity 1.5 cm/s
Wavelength: 8 sec
Mimicking Breathing signal
2 min 30 sec
Flow velocity 2 cm/s
Wavelength: 8 sec
Mimicking Breathing signal
2 min 30 sec
Flow velocity 2.5 cm/s
Wavelength: 8 sec
Mimicking Breathing signal
2 min 30 sec
No Flow
Repeated total 1 cycles
1 cycle of phantom experiment
2 min 30 sec
Flow velocity 1 cm/s
Wavelength: 1 sec
Mimicking Cardiac pulsation
2 min 30 sec
Flow velocity 2 cm/s
Wavelength: 1 sec
Mimicking Cardiac pulsation
2 min 30 sec
Flow velocity 3 cm/s
Wavelength: 1 sec
Mimicking Cardiac pulsation
2 min 30 sec
Flow velocity 1.5 cm/s
Wavelength: 8 sec
Mimicking Breathing signal
2 min 30 sec
Flow velocity 2 cm/s
Wavelength: 8 sec
Mimicking Breathing signal
2 min 30 sec
Flow velocity 2.5 cm/s
Wavelength: 8 sec
Mimicking Breathing signal
e
Magnitude image
Real-time phase contrast
f
Segment ROI
64 x 64 boundary box
Extract voxels related to cardiac & breathing
Extract mask
Obtain flow signal from mask
g
(cm/s)
No Flow
Flow signal
Flow velocity
4
3
2
1
0
−1
0
20
40
60
80
100
120
140
Time (sec)

Figure S17. Experimental setup using a flow phantom to simulate CSF motion. a, Schematic illustration of the flow phantom system designed to mimic human CSF dynamics. Arrows indicate flow direction within the phantom. b, Photograph of the experimental setup showing a programmable motor system connected to internal tubing embedded in the phantom to generate pulsatile water flow simulating CSF motion. c, Representative MPRAGE image of the flow phantom for anatomical reference. d, Experimental protocol. The phantom was programmed to simulate both cardiac-like and respiratory-like CSF oscillations. Each run included six distinct flow conditions, repeated across three cycles. After completion of the second cycle, a no-flow condition was introduced before the final cycle to serve as a baseline control for signal comparison. e, Representative PC-MRI images from the flow phantom experiment, including magnitude and phase-contrast images. f, ROIs were defined by segmenting flow areas from the phase images. Binary masks were generated, and mean signal values from both the flow region and surrounding overlay region were extracted for analysis. g, PC-MRI signal under the no-flow condition, serving as a baseline. Abbreviation: MPRAGE, magnetization-prepared rapid gradient-echo; ROI, region-of-interest.

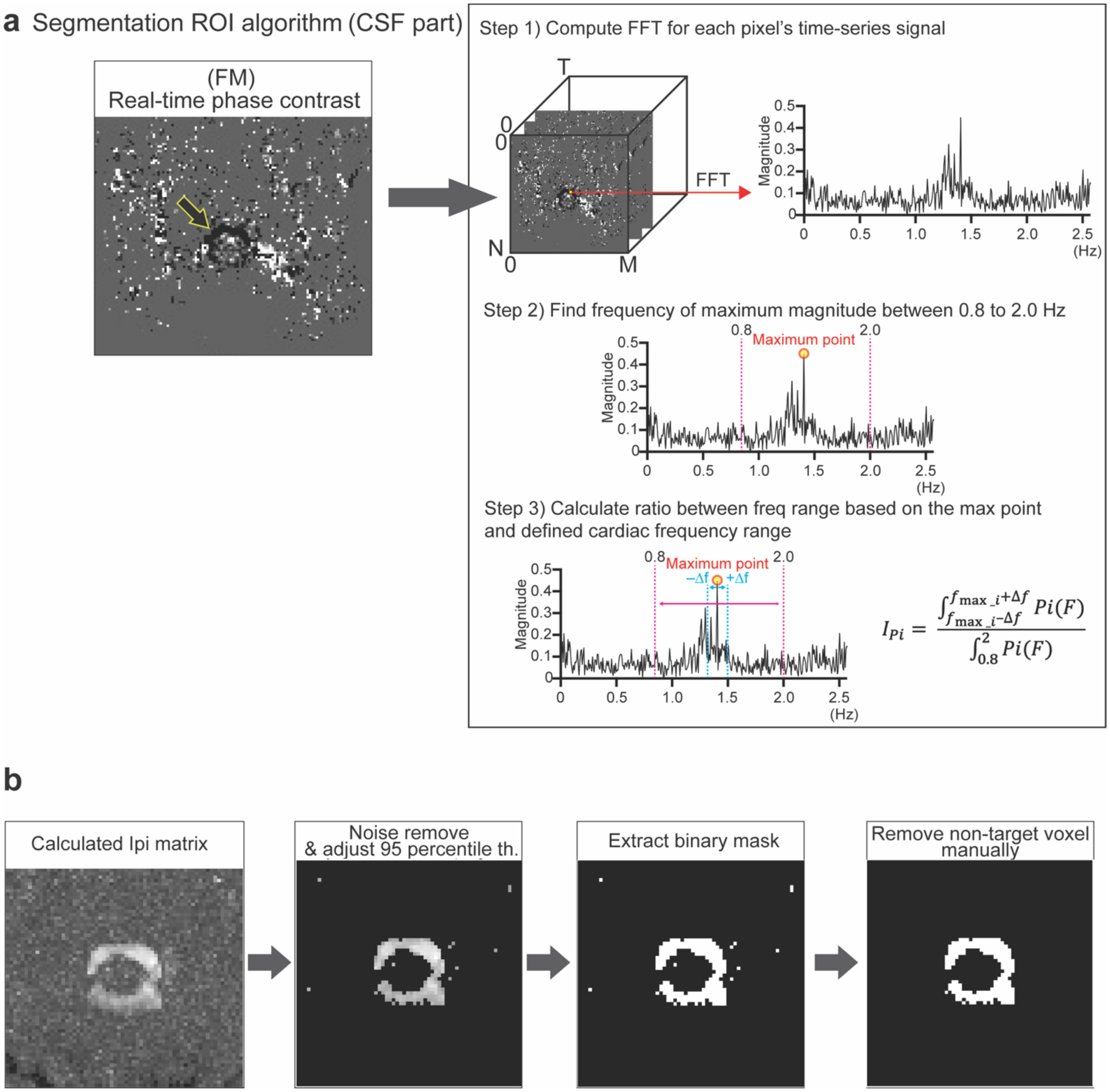

Figure S18. Segmentation of CSF flow regions in PC-MRI. a, To identify CSF flow regions, a voxel-wise FFT was performed on the time-resolved PC-MRI images. For each voxel, the maximum spectral magnitude was detected within a predefined frequency range of 0.8–2.0 Hz. The intensity ratio was calculated by integrating the power spectrum within this delta frequency window and normalizing it according to a predefined formula, reflecting the relative spectral contribution of CSF flow. b, The resulting intensity projection image (Ipi matrix) was denoised and sorted to apply a 95th percentile threshold, generating a binary mask that isolated CSF-dominant voxels. Manual corrections were performed to remove residual non-CSF voxels not

adequately excluded by thresholding**.** Abbreviation: CSF, cerebrospinal fluid; FFT, fast fourier transform; MRI, magnetic resonance imaging; PCMRI; phase-contrast MRI; ROI, region-of-interest.

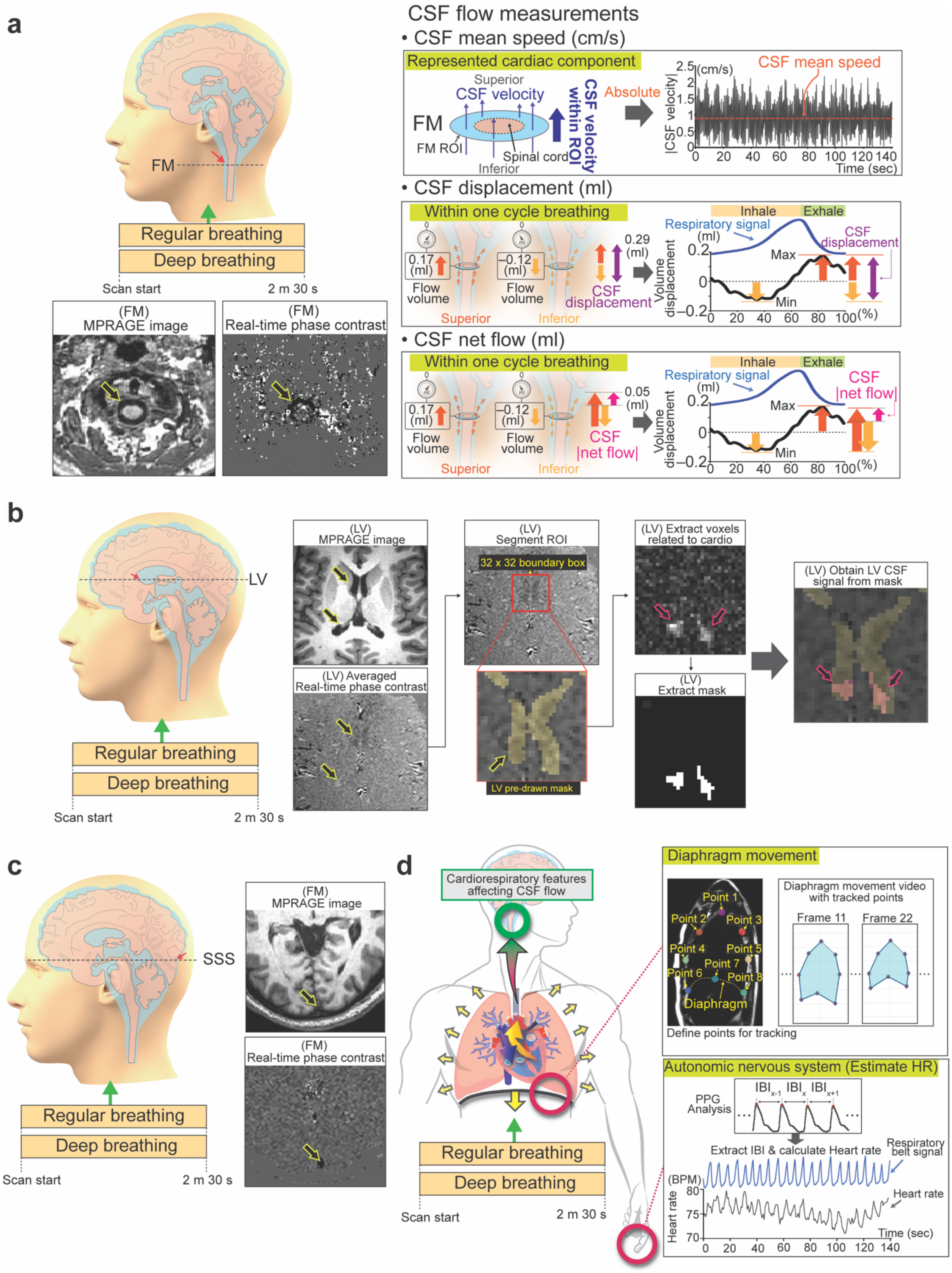
a
FM
Regular breathing
Deep breathing
Scan start
2 m 30 s
(FM) MPRAGE image
(FM) Real-time phase contrast
CSF flow measurements
• CSF mean speed (cm/s)
Represented cardiac component
Superior
CSF velocity
CSF velocity within ROI
FM
FM ROI
Spinal cord
Inferior
Absolute
CSF mean speed
|CSF velocity| (cm/s)
Time (sec)
• CSF displacement (ml)
Within one cycle breathing
0.17 (ml)
-0.12 (ml)
Flow volume
0.29 (ml)
CSF displacement
Superior
Inferior
Inhale
Exhale
Respiratory signal
Volume displacement (ml)
Max
Min
(%)
• CSF net flow (ml)
Within one cycle breathing
0.05 (ml)
CSF |net flow|
b
LV
(LV) MPRAGE image
(LV) Averaged Real-time phase contrast
(LV) Segment ROI
32 x 32 boundary box
LV pre-drawn mask
(LV) Extract voxels related to cardio
(LV) Extract mask
(LV) Obtain LV CSF signal from mask
c
SSS
(FM) MPRAGE image
(FM) Real-time phase contrast
d
Cardiorespiratory features affecting CSF flow
Diaphragm movement
Point 1
Point 2
Point 3
Point 4
Point 5
Point 6
Point 7
Point 8
Diaphragm
Define points for tracking
Diaphragm movement video with tracked points
Frame 11
Frame 22
Autonomic nervous system (Estimate HR)
PPG Analysis
IBI x-1 IBI x IBI x+1
Extract IBI & calculate Heart rate
Respiratory belt signal
Heart rate (BPM)
Heart rate
Time (sec)

Figure S19. Schematic of the brain showing the CSF measurement location at the FM. a, The illustration show how three representative CSF flow features are calculated: CSF mean speed, CSF displacement, and CSF net flow. b, Schematic of the brain showing CSF measurement locations in the LV. Manually drawn anterior LV ROI (yellow) did not show statistical group differences. Using automatic detection of the cardiac component, the entrance of the foramen of Monro was identified as a detectable ROI for analysis (red). c, Schematic of the brain showing the venous measurement location at the SSS. d, Chest and diaphragm movement were quantified as mechanical features of respiration using single-shot T2-weighted MRI images and a motion-tracking deep learning software. Individual lung areas were normalized for comparison. As an autonomic feature, cardiac HR was extracted from PPG recordings acquired during PC-MRI. Abbreviation: BPM, beats per minute; CSF, cerebrospinal fluid; DB, deep breathing; FM, foramen magnum; HR, heart rate; IBI, inter-beat interval; LV, lateral ventricle; MPRAGE, magnetization-prepared rapid gradient-echo; NT, non-trained; PPG, photoplethysmography; RB, regular breathing; SSS, superior sagittal sinus; T, trained; ROI, region-of-interest.

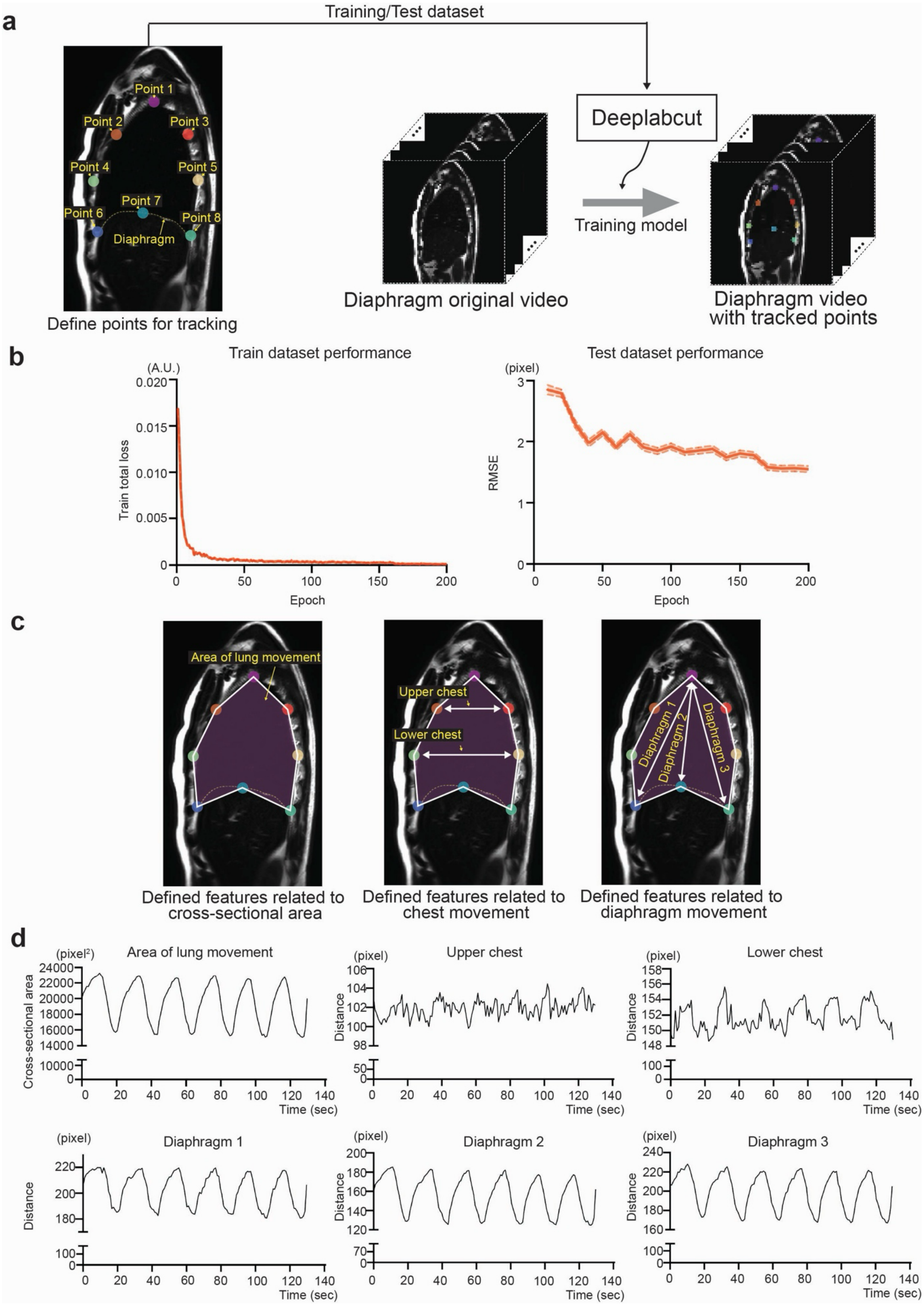

a
Training/Test dataset
Point 1
Point 2
Point 3
Point 4
Point 5
Point 6
Point 7
Point 8
Diaphragm
Define points for tracking
Diaphragm original video
Deeplabcut
Training model
Diaphragm video with tracked points
b
Train dataset performance
(A.U.)
Train total loss
Epoch
Test dataset performance
(pixel)
RMSE
Epoch
c
Area of lung movement
Defined features related to cross-sectional area
Upper chest
Lower chest
Defined features related to chest movement
Diaphragm 1
Diaphragm 2
Diaphragm 3
Defined features related to diaphragm movement
d
Area of lung movement
(pixel²)
Cross-sectional area
Time (sec)
Upper chest
(pixel)
Distance
Lower chest
Diaphragm 1
Diaphragm 2
Diaphragm 3

Figure S20. Quantitative analysis of diaphragm movement using deep learning-based tracking. a, Quantitative tracking of diaphragm motion was performed on the T2 diaphragm image using DeepLabCut, a deep learning-based framework for markerless pose estimation. This approach enables automated tracking of predefined anatomical landmarks across respiratory video frames. b, The model was separately trained on 90 recordings acquired from 45 individuals during both RB and DB conditions (n = 90). The model achieved high tracking accuracy, converging to an average test RMSE of approximately 1.5 pixels. c, Six quantitative features of diaphragm movement were extracted from the tracked points. "Area of lung movement" was defined as the enclosed cross-sectional area formed by tracked points. “Upper chest” and “Lower chest” metrics were calculated as the Euclidean distances between each paired point on the chest wall. “Diaphragm 1,” “Diaphragm 2,” and “Diaphragm 3” were also defined based on Euclidean distances between anatomical landmarks on the diaphragm. d, The extracted features captured distinct patterns of diaphragmatic motion across the respiratory cycle. The illustrated dataset demonstrates a pattern observed during DB. Abbreviation: A.U., arbitrary unit.
