# Supplementary material for: Human cerebrospinal fluid net flow enhanced by respiration during the awake state
Source: Nat Commun. 2025 Dec 13;16:11499. doi: 10.1038/s41467-025-66548-4 (PMC12749403; doi:10.1038/s41467-025-66548-4)
Supplement: Supplementary file 2 — Description of Additional Supplementary Files [file 41467_2025_66548_MOESM2_ESM.pdf]

Description of Additional Supplementary Files

File name: Supplementary Movie 1

Description: Representative case from a non-trained participant showing MRI-overlaid modeled diaphragm movement during regular breathing.

File name: Supplementary Movie 2

Description: Representative case from a non-trained participant showing modeled diaphragm movement during regular breathing.

File name: Supplementary Movie 3

Description: Representative case from a non-trained participant showing MRI-overlaid modeled diaphragm movement during deep breathing.

File name: Supplementary Movie 4

Description: Representative case from a non-trained participant showing modeled diaphragm movement during deep breathing.

File name: Supplementary Movie 5

Description: Representative case from a trained participant showing MRI-overlaid modeled diaphragm movement during regular breathing.

File name: Supplementary Movie 6

Description: Representative case from a trained participant showing modeled diaphragm movement during regular breathing.

File name: Supplementary Movie 7

Description: Representative case from a trained participant showing MRI-overlaid modeled diaphragm movement during deep breathing.

File name: Supplementary Movie 8

Description: Representative case from a trained participant showing modeled diaphragm movement during deep breathing.
