# Supplementary material for: Human cerebrospinal fluid net flow enhanced by respiration during the awake state
Source: Nat Commun. 2025 Dec 13;16:11499. doi: 10.1038/s41467-025-66548-4 (PMC12749403; doi:10.1038/s41467-025-66548-4)
Supplement: Supplementary file 11 — Reporting Summary [file 41467_2025_66548_MOESM11_ESM.pdf]

# nature portfolio

Corresponding author(s): Paul H. Min, Petrice M. Cogswell

Last updated by author(s): 10/7/2025

# Reporting Summary

Nature Portfolio wishes to improve the reproducibility of the work that we publish. This form provides structure for consistency and transparency in reporting. For further information on Nature Portfolio policies, see our Editorial Policies and the Editorial Policy Checklist.

## Statistics

For all statistical analyses, confirm that the following items are present in the figure legend, table legend, main text, or Methods section.

| n/a | Confirmed | |
|---|---|---|
| ☐ | ☒ | The exact sample size (*n*) for each experimental group/condition, given as a discrete number and unit of measurement |
| ☐ | ☒ | A statement on whether measurements were taken from distinct samples or whether the same sample was measured repeatedly |
| ☐ | ☒ | The statistical test(s) used AND whether they are one- or two-sided<br>*Only common tests should be described solely by name; describe more complex techniques in the Methods section.* |
| ☐ | ☒ | A description of all covariates tested |
| ☐ | ☒ | A description of any assumptions or corrections, such as tests of normality and adjustment for multiple comparisons |
| ☐ | ☒ | A full description of the statistical parameters including central tendency (e.g. means) or other basic estimates (e.g. regression coefficient) AND variation (e.g. standard deviation) or associated estimates of uncertainty (e.g. confidence intervals) |
| ☐ | ☒ | For null hypothesis testing, the test statistic (e.g. *F*, *t*, *r*) with confidence intervals, effect sizes, degrees of freedom and *P* value noted<br>*Give P values as exact values whenever suitable.* |
| ☒ | ☐ | For Bayesian analysis, information on the choice of priors and Markov chain Monte Carlo settings |
| ☐ | ☒ | For hierarchical and complex designs, identification of the appropriate level for tests and full reporting of outcomes |
| ☐ | ☒ | Estimates of effect sizes (e.g. Cohen's *d*, Pearson's *r*), indicating how they were calculated |

*Our web collection on statistics for biologists contains articles on many of the points above.*

## Software and code

Policy information about availability of computer code

| | |
|---|---|
| Data collection | Matlab, R |
| Data analysis | Custom code used in this study is available from the corresponding author upon reasonable request. No external software beyond standard analysis tools was used. |

For manuscripts utilizing custom algorithms or software that are central to the research but not yet described in published literature, software must be made available to editors and reviewers. We strongly encourage code deposition in a community repository (e.g. GitHub). See the Nature Portfolio guidelines for submitting code & software for further information.

## Data

Policy information about availability of data

All manuscripts must include a data availability statement. This statement should provide the following information, where applicable:

- Accession codes, unique identifiers, or web links for publicly available datasets
- A description of any restrictions on data availability
- For clinical datasets or third party data, please ensure that the statement adheres to our policy

The data supporting the findings of this study are available from the corresponding author upon reasonable request. Processed data used in the analyses are available within the Article and its Supplementary Information.

## Research involving human participants, their data, or biological material

Policy information about studies with human participants or human data. See also policy information about sex, gender (identity/presentation), and sexual orientation and race, ethnicity and racism.

| | |
|---|---|
| Reporting on sex and gender | Sex and Age are match between each group. |
| Reporting on race, ethnicity, or other socially relevant groupings | NA |
| Population characteristics | Physiological covariates (age, sex, blood pressure, and respiratory rate) were assessed |
| Recruitment | Local participants were recruited by flyers and local contact. |
| Ethics oversight | Mayo Clinic Institutional Review Board |

Note that full information on the approval of the study protocol must also be provided in the manuscript.

# Field-specific reporting

Please select the one below that is the best fit for your research. If you are not sure, read the appropriate sections before making your selection.

[x] Life sciences [ ] Behavioural & social sciences [ ] Ecological, evolutionary & environmental sciences

For a reference copy of the document with all sections, see nature.com/documents/nr-reporting-summary-flat.pdf

# Life sciences study design

All studies must disclose on these points even when the disclosure is negative.

| | |
|---|---|
| Sample size | T n=20, NT n=25 |
| Data exclusions | Healthy participants were defined as having no medical conditions affecting brain function, concentration, memory, balance, or coordination |
| Replication | NA |
| Randomization | NA |
| Blinding | NA |

# Reporting for specific materials, systems and methods

We require information from authors about some types of materials, experimental systems and methods used in many studies. Here, indicate whether each material, system or method listed is relevant to your study. If you are not sure if a list item applies to your research, read the appropriate section before selecting a response.

## Materials & experimental systems

| n/a | Involved in the study | |
|---|---|---|
| [x] | [ ] | Antibodies |
| [x] | [ ] | Eukaryotic cell lines |
| [x] | [ ] | Palaeontology and archaeology |
| [x] | [ ] | Animals and other organisms |
| [x] | [ ] | Clinical data |
| [x] | [ ] | Dual use research of concern |
| [x] | [ ] | Plants |

## Methods

| n/a | Involved in the study | |
|---|---|---|
| [x] | [ ] | ChIP-seq |
| [x] | [ ] | Flow cytometry |
| [ ] | [x] | MRI-based neuroimaging |

# Plants

| | |
|---|---|
| Seed stocks | *Report on the source of all seed stocks or other plant material used. If applicable, state the seed stock centre and catalogue number. If plant specimens were collected from the field, describe the collection location, date and sampling procedures.* |
| Novel plant genotypes | *Describe the methods by which all novel plant genotypes were produced. This includes those generated by transgenic approaches, gene editing, chemical/radiation-based mutagenesis and hybridization. For transgenic lines, describe the transformation method, the number of independent lines analyzed and the generation upon which experiments were performed. For gene-edited lines, describe the editor used, the endogenous sequence targeted for editing, the targeting guide RNA sequence (if applicable) and how the editor was applied.* |
| Authentication | *Describe any authentication procedures for each seed stock used or novel genotype generated. Describe any experiments used to assess the effect of a mutation and, where applicable, how potential secondary effects (e.g. second site T-DNA insertions, mosiacism, off-target gene editing) were examined.* |

# Magnetic resonance imaging

## Experimental design

| | |
|---|---|
| Design type | Resting |
| Design specifications | NA |
| Behavioral performance measures | NA |

## Acquisition

| | |
|---|---|
| Imaging type(s) | Real-Time 2D Phase-Contrast MRI Acquisition |
| Field strength | 3T |
| Sequence & imaging parameters | The parameters for the real-time PC-MRI included a repetition time/echo time of 102/46 ms, a flip angle of 30 degrees, a field of view of 217 × 217 mm², a matrix size of 172 × 169, spatial resolution of 1.26 × 1.28 mm², slice thickness of 3 mm, temporal resolution of 0.19 seconds, sensitivity encoding (Ry = 3), and a total scan time of 140 seconds. |
| Area of acquisition | FM, 4V, AQ, LV, SSS |

Diffusion MRI ☐ Used ☒ Not used

## Preprocessing

| | |
|---|---|
| Preprocessing software | Matlab |
| Normalization | Intensity normalization |
| Normalization template | NA |
| Noise and artifact removal | Detrending (first order) |
| Volume censoring | NA |

## Statistical modeling & inference

| | |
|---|---|
| Model type and settings | R |
| Effect(s) tested | ANOVA and post-hoc t-test |

Specify type of analysis: ☐ Whole brain ☒ ROI-based ☐ Both

Anatomical location(s) FM, LV, SSS

| | |
|---|---|
| Statistic type for inference (See Eklund et al. 2016) | ROI analysis |
| Correction | Pearson Correlation |

## Models & analysis

n/a | Involved in the study

- [x] [ ] Functional and/or effective connectivity
- [x] [ ] Graph analysis
- [ ] [x] Multivariate modeling or predictive analysis

| | |
|---|---|
| Multivariate modeling and predictive analysis | To assess causality and directional relationships among these variables, we applied both multivariate regression and structural equation modeling (SEM). SEM was used to identify feature interrelationships and directional effects, while multivariate regression estimated the unique contribution of each variable. We further expanded the features and categorizing variables into three groups: respiratory features (e.g., timing parameters, maximum and minimum values, diaphragm and chest displacement), physiological features (e.g., timing parameters, maximum and minimum values, SSS and HR displacement), and CSF flow features (e.g., timing parameters, maximum and minimum values, flow, velocity, displacement, and net flow). |
