# Supplementary material for: Author Correction: Human cerebrospinal fluid net flow enhanced by respiration during the awake state
Source: Nat Commun. 2026 Feb 5;17:1393. doi: 10.1038/s41467-026-69399-9 (PMC12877048; doi:10.1038/s41467-026-69399-9)
Supplement: Supplementary file 1 — List of edits to original article [file 41467_2026_69399_MOESM1_ESM.pdf]

**Supplementary Information to Author Correction: Human cerebrospinal fluid net flow enhanced by respiration during the awake state**

Correction to: *Nature Communications* https://doi.org/10.1038/s41467-025-66548-4, published online 13 December 2025.

**LIST OF CHANGES TO ARTICLE**

**1. Formatting update for variance reporting (SD/SEM)**
Reason for correction: In one paragraph, SD and SEM were used inconsistently. We standardized all variance reporting to mean ± SD. Mean values, *p*-values, and statistical significance remain unchanged.
Location: Results section, 4th paragraph

Original version:
"Group comparisons are summarized in Fig. 1. During RB at the FM, T showed higher CSF mean peak velocity (T 1.00 ± 0.24 cm/s; NT 0.78 ± 0.20 cm/s; p = 0.0019), higher CSF mean speed (T 0.83 ± 0.05 cm/s; NT 0.66 ± 0.03 cm/s; p = 0.0059) and higher CSF displacement (T 0.25 ± 0.04 ml; NT 0.09 ± 0.01 ml; p = 0.0005), with a trend toward higher CSF net flow (T 33.33 ± 9.48 µl; NT 15.46 ± 2.99 µl; p = 0.0573). During RB at the LV, T showed no difference in CSF mean peak velocity (T 0.21 ± 0.07 cm/s; NT 0.19 ± 0.05 cm/s; p = 0.3238) but higher values across the other three measures: CSF mean speed (T 0.19 ± 0.02 cm/s; NT 0.15 ± 0.01 cm/s; p = 0.0411), CSF displacement (T 0.049 ± 0.012 ml; NT 0.018 ± 0.003 ml; p = 0.0093), and CSF net flow (T 10.78 ± 2.75 µl; NT 4.25 ± 1.07 µl; p = 0.0229) (Fig. 1a). During DB at the FM, T showed higher values across all four measures: CSF mean peak velocity (T 1.02 ± 0.29 cm/s; NT 0.77 ± 0.23 cm/s; p = 0.0022), CSF mean speed (T 0.84 ± 0.06 cm/s; NT 0.61 ± 0.03 cm/s; p = 0.0013), CSF displacement (T 0.59 ± 0.07 ml; NT 0.22 ± 0.03 ml; p < 0.0001), and CSF net flow (T 70.28 ± 12.95 µl; NT 37.28 ± 5.69 µl; p = 0.0172). During DB at the LV, CSF mean peak velocity (T 0.21 ± 0.07 cm/s; NT 0.16 ± 0.05 cm/s; p = 0.0135) and CSF mean speed was significantly higher in T (T 0.19 ± 0.02 cm/s; NT 0.14 ± 0.01 cm/s; p = 0.0176), while CSF displacement and CSF net flow showed no group differences (Fig. 1b)."

Corrected version (changes listed in boldface):
"Group comparisons are summarized in Fig. 1. During RB at the FM, T showed higher CSF mean peak velocity (T 1.00 ± 0.24 cm/s; NT 0.78 ± 0.20 cm/s; p = 0.0019), higher CSF mean speed (T 0.83 ± **0.22** cm/s; NT 0.66 ± **0.18** cm/s; p = 0.0059) and higher CSF displacement (T 0.25 ± **0.20** ml; NT 0.09 ± **0.07** ml; p = 0.0005), with a trend toward higher CSF net flow (T 33.33 ± **42.41** µl; NT 15.46 ± **15.55** µl; p = 0.0573). During RB at the LV, T showed no difference in CSF mean peak velocity (T 0.21 ± 0.07 cm/s; NT 0.19 ± 0.05 cm/s; p = 0.3238) but higher values across the other three measures: CSF mean speed (T 0.19 ± **0.07** cm/s; NT 0.15 ± **0.04** cm/s; p = 0.0411), CSF displacement (T 0.049 ± **0.052** ml; NT 0.018 ± **0.014** ml; p = 0.0093), and CSF net flow (T 10.78 ± **12.01** µl; NT 4.25 ± **5.13** µl; p = 0.0229) (Fig. 1a). During DB at the FM, T showed higher values across all four measures: CSF mean peak velocity (T 1.02 ± 0.29 cm/s; NT 0.77 ± 0.23 cm/s; p = 0.0022), CSF mean speed (T 0.84 ± **0.27** cm/s; NT 0.61 ± **0.18** cm/s; p = 0.0013), CSF displacement (T 0.59 ± **0.32** ml; NT 0.22 ± **0.16** ml; p < 0.0001), and CSF net flow (T 70.28 ± **57.91** µl;

NT 37.28 ± **29.59** μl; p = 0.0172). During DB at the LV, CSF mean peak velocity (T 0.21 ± 0.07 cm/s; NT 0.16 ± 0.05 cm/s; p = 0.0135) and CSF mean speed was significantly higher in T (T 0.19 ± **0.07** cm/s; NT 0.14 ± **0.04** cm/s; p = 0.0176), while CSF displacement and CSF net flow showed no group differences (Fig. 1b)."

**2. Correction to reported NT $R^2$ value**
Reason for correction: The NT $R^2$ value for the correlation was reported incorrectly and has been updated to match the validated analysis (0.1751 → 0.0900). The *p*-value and statistical significance remain unchanged.
Location: Results section, 12th paragraph.

Original version:
"In the LV, SSS displacement also correlated with CSF displacement (T $R^2$= 0.4181, p < 0.0001; NT $R^2$ = 0.1751, p = 0.0428) and showed correlation with CSF net flow only with T (T $R^2$ = 0.1823, p = 0.0075; NT $R^2$ = 0.0057, p = 0.6175), whereas HR displacement did not correlate with any CSF flow features (Fig. S12b).

Corrected version (changes listed in boldface):
"In the LV, SSS displacement also correlated with CSF displacement (T $R^2$ = 0.4181, p < 0.0001; NT $R^2$ **= 0.0900**, p = 0.0428) and showed correlation with CSF net flow only with T (T $R^2$ = 0.1823, p = 0.0075; NT $R^2$ = 0.0057, p = 0.6175), whereas HR displacement did not correlate with any CSF flow features (Fig. S12b)."

**3. Unit formatting update (net flow and displacement)**
Reason for correction: Net flow should be reported in μl (not mL), and displacement should be written as ml (not mL).
Location: Results section, 16th paragraph.

Original version:
"The NT participant (male, 62 yr) showed an increase in CSF mean speed (0.59 → 0.83 cm/s), displacement (0.10 → 0.21 mL), and net flow (20.27 → 64.72 mL) during DB. The T participant (male, 59 yr) exhibited greater overall CSF dynamics, with mean speed (0.89 → 1.05 cm/s) and displacement (0.30 → 1.32 mL) increasing during DB, while net flow varied (48.59 → 24.64 mL)."

Corrected version (changes listed in boldface):
"The NT participant (male, 62 yr) showed an increase in CSF mean speed (0.59 → 0.83 cm/s), displacement (0.10 → 0.21 **ml**), and net flow (20.27 → 64.72 **μl**) during DB. The T participant (male, 59 yr) exhibited greater overall CSF dynamics, with mean speed (0.89 → 1.05 cm/s) and displacement (0.30 → 1.32 **ml**) increasing during DB, while net flow varied (48.59 → 24.64 **μl**)."

**4. Update to participant counts in the High group**
Reason for correction: The High group sample sizes were reported incorrectly and were updated to reflect the correct participant numbers for each group. The results are unchanged.
Location: Results section, 17th paragraph.

Original version:
”At the FM, the High group included participants across both groups and breathing conditions (8 T-RB, 16 T-DB, 4 NT-RB, 14 NT-DB, out of 19 in each condition), and at the LV, the High group similarly included a mixture of participants (12 T-RB, 12 T-DB, 7 NT-RB, 11 NT-DB).”

Corrected version (changes listed in boldface):
“At the FM, the High group included participants across both groups and breathing conditions (**9 T-RB, 17 T-DB, 4 NT-RB, 15 NT-DB, out of 20 in T and 25 in NT for each breathing condition**), and at the LV, the High group similarly included a mixture of participants (**8 T-RB, 16 T-DB, 4 NT-RB, 13 NT-DB, out of 19 in T and 23 in NT for each breathing condition**).”

**5. Correction to unit label for chest displacement**
Reason for correction: The unit for chest displacement was written incorrectly as “sec A.U.” for the Low group; it should be “A.U.”.
Location: Results section, 17th paragraph.

Original version:
“Participants in the High group showed significantly longer inhale length (High 7.999 ± 4.701 sec; Low 3.857 ± 2.161 sec; $p < 0.0001$), greater chest displacement (High 0.0326 ± 0.0238 A.U.; Low 0.0242 ± 0.0156 sec A.U.; $p = 0.0497$), and greater diaphragm displacement (High 0.3935 ± 0.1725 A.U.; Low 0.2085 ± 0.1330 A.U.; $p < 0.0001$) compared with those in the Low.”

Corrected version (changes listed in boldface):
“Participants in the High group showed significantly longer inhale length (High 7.999 ± 4.701 sec; Low 3.857 ± 2.161 sec; $p < 0.0001$), greater chest displacement (High 0.0326 ± 0.0238 A.U.; Low 0.0242 ± 0.0156 **A.U**.; $p = 0.0497$), and greater diaphragm displacement (High 0.3935 ± 0.1725 A.U.; Low 0.2085 ± 0.1330 A.U.; $p < 0.0001$) compared with those in the Low.”

**6. Correction to a typographical error and associated p-value**
Reason for correction: There was a typo in the SD value (0.0.2354 → 0.2354), and the *p*-value for FM mean peak velocity was incorrect, so it was updated ($p < 0.0001$ → $p = 0.0366$). Mean values and statistical significance remain unchanged.
Location: Results section, 17th paragraph.

Original version:
”Higher FM net flow was also associated with greater FM CSF mean peak velocity (High 0.9347 ± 0.2739 cm/s; Low 0.8204 ± 0.0.2354 cm/s; $p < 0.0001$), FM CSF displacement (High 0.4088 ± 0.2973 ml; Low 0.1369 ± 0.1386 ml; $p < 0.0001$) and with increased LV CSF displacement (High 0.0721 ± 0.057 ml; Low 0.0304 ± 0.0327 ml; $p < 0.0001$) and net flow (High 9.809 ± 10.02 µl; Low 5.678 ± 8.881 µl; $p = 0.0486$)."

Corrected version (changes listed in boldface):
“Higher FM net flow was also associated with greater FM CSF mean peak velocity (High 0.9347 ± 0.2739 cm/s; Low 0.8204 ± **0.2354 cm/s; p = 0.0366**), FM CSF displacement (High 0.4088 ± 0.2973 ml; Low 0.1369 ± 0.1386 ml; $p < 0.0001$) and with increased LV CSF displacement (High 0.0721 ± 0.057 ml; Low 0.0304 ± 0.0327 ml; $p < 0.0001$) and net flow (High 9.809 ± 10.02 µl; Low 5.678 ± 8.881 µl; $p = 0.0486$)."

**7. Update to supplementary figure references**
Reason for correction: The supplementary figure numbers were incorrect and were updated to the correct ones (Fig. S2a/S2d → Fig. S17a/S17d).
Location: Methods section, 2nd paragraph.

Original version:
"This test examined the influence of flow directionality on the measured PC-MRI signal by alternating the flow direction while maintaining identical velocity and frequency parameters (Fig. S2a).
In the first test, six sessions were conducted by driving the motor system at sinusoidal velocities of 1, 2, and 3 cm/s with a cycle length of 1 s (cardiac pulsation), and 1.5, 2, and 2.5 cm/s with a cycle length of 8 s (respiratory pulsation). As shown in Fig. S2d, each of the six sessions was repeated twice with a 2 min 30 s interval, followed by a 2 min 30 s no-flow period."

Corrected version (changes listed in boldface):
"This test examined the influence of flow directionality on the measured PC-MRI signal by alternating the flow direction while maintaining identical velocity and frequency parameters (**Fig. S17a**).
In the first test, six sessions were conducted by driving the motor system at sinusoidal velocities of 1, 2, and 3 cm/s with a cycle length of 1 s (cardiac pulsation), and 1.5, 2, and 2.5 cm/s with a cycle length of 8 s (respiratory pulsation). As shown in **Fig. S17d,** each of the six sessions was repeated twice with a 2 min 30 s interval, followed by a 2 min 30 s no-flow period."
